# eIF2A regulates cell migration in a translation-independent manner

**DOI:** 10.1126/sciadv.adu5668

**Published:** 2025-08-01

**Authors:** Jennifer Jungfleisch, Neus Mestre-Farràs, Raúl Gómez-Riera, Oriane Pourcelot, Edouard Bertrand, Nadia Halidi, Fátima Gebauer

**Affiliations:** ^1^ Centre for Genomic Regulation (CRG), The Barcelona Institute of Science and Technology (BIST), Dr. Aiguader 88, Barcelona 08003, Spain.; ^2^ Institute of Human Genetics, University of Montpellier, CNRS, Montpellier, France.; ^3^ Universitat Pompeu Fabra (UPF), Barcelona 08003, Spain.

## Abstract

The RNA-binding protein eukaryotic translation initiation factor 2A (eIF2A) is an alternative translation initiation factor shown to drive tumor formation by facilitating translation from near-cognate initiation codons. Here, we uncover a function for eIF2A in regulating cell migration in a manner independent of overt control of translation. Using a melanoma cell model consisting of nontumoral melanocytic Mel-ST cells and their metastatic counterpart obtained by *H-Ras* transformation, we unexpectedly find minimal effects of eIF2A depletion on translation. Interactome studies identified centrosomal proteins as major binding partners of eIF2A. We found that eIF2A colocalizes with the centrosome, enhances centrosome composition, and promotes centrosome orientation during cell migration. Migration requires the C-terminal disordered region of eIF2A, involved in mRNA binding. Interaction with mRNA, however, does not require ongoing translation. These findings reveal a role for eIF2A in centrosome dynamics beyond its traditional function in translation.

## Teaser

Findings show that eIF2A controls cell migration by regulating centrosome content beyond its role in translation.

## INTRODUCTION

Protein synthesis is a fundamental cellular process that allows cells to fine-tune their responses and adapt to various stimuli and environmental changes. This capability is particularly crucial in the context of cancer, as cancer cells need to modulate protein synthesis to drive their aggressive behavior and survival under diverse conditions. Among the various stages of protein synthesis, translation initiation has emerged as the most highly regulated step. Traditionally, canonical translation initiation, which relies on the eukaryotic translation initiation factor 2 α (eIF2α), has been the focus of research. However, recent investigations have begun to uncover the growing importance of alternative initiation mechanisms in cancer biology (*1*, *2*).

One such pathway involves the alternative initiation factor 2A (eIF2A). eIF2A has been mainly implicated in translation initiation under stress conditions, where the canonical factor eIF2α is inactivated by phosphorylation, and eIF2A takes over in binding the initiator tRNA and promoting initiation codon recognition. eIF2A has been shown to promote translation from upstream open reading frames (uORFs) by recruiting the initiator methionyl–tRNA (tRNA_i_) to AUG start codons or leucyl-tRNA_i_ to CUG and UUG near-cognate codons (*3*–*5*), from cellular internal ribosome entry sites (IRES) (*6*–*8*) and from viral RNAs such as hepatitis C virus (HCV) and Sindbis virus or enterovirus (*7*, *9*, *10*) [reviewed in (*11*)]. Conflicting literature, however, has emerged, suggesting that eIF2A does not promote translation of HCV or Sindbis viral mRNA (*12*–*14*), that most alternative translation under stress depends on eIF2α (*15*), or that eIF2A has little to no role in translation initiation in yeast (*16*). One study performed using in vitro translation assays even suggested that eIF2A inhibits translation by sequestering 40*S* ribosomal subunits (*17*). Hence, while it seems clear that eIF2A does not function in canonical translation initiation, its exact function in alternative translation is controversial and might be context dependent.

Context-dependent translation regulation plays a major role in cancer progression, and several observations link eIF2A to cancer. The *EIF2A* locus is amplified in patients with lung and head and neck squamous cell carcinoma (SCC) as well as esophageal carcinoma (*3*), and elevated eIF2A levels have been associated with poor prognosis in breast cancer (*18*). Furthermore, eIF2A promotes breast cancer cell survival during paclitaxel-mediated integrated stress response (*18*) and stimulates translation of *CCNB1* mRNA leading to hepatocellular carcinoma progression (*19*). In addition, eIF2A promotes translation from an upstream CUG in *PTEN* mRNA, leading to an N-terminally extended form of PTEN called PTENα that participates in mitochondrial energy metabolism and supports the energetic demand of rapidly proliferating cancer cells (*20*). Last, eIF2A-mediated uORF translation plays an essential role in SCC initiation and progression (*3*). These reports point toward an important function of eIF2A as an alternative translation initiation factor contributing to cancer cell adaptation to the varying conditions encountered during malignant progression.

Metastatic melanoma, an aggressive form of skin cancer with poor prognosis, presents an intriguing context for investigating the potential functions of eIF2A. Melanoma is characterized by its high metastatic potential and resistance to conventional therapies (*21*). The ability of melanoma cells to adapt to various stress conditions, such as nutrient deprivation and hypoxia, is crucial for their survival and metastatic spread (*22*). In a previous screen, we had found eIF2A as a factor potentially involved in melanoma metastatic progression (*23*). Here, we explore the functions of eIF2A during oncogenic transformation using a melanoma cell model consisting of the nontumoral melanocytic cell line Mel-ST and its metastatic counterpart Mel-STR obtained by H-Ras^G12V^ overexpression (*24*). This system allows focused analysis of changes caused by metastatic transformation while minimizing confounding effects from the heterogeneity of melanoma cells. We show that oncogenic transformation leads to acquired dependencies on eIF2A for tumoral traits including spheroid growth and migration. Unexpectedly, translatome and transcriptome analysis indicate that these dependencies do not rely on the function of eIF2A in translation. A focus on migration revealed that they depend on eIF2A’s RNA-binding activity and on a function of eIF2A in enforcing centrosome composition and orientation.

## RESULTS

### eIF2A promotes tumoral traits

**Fig. 1.**
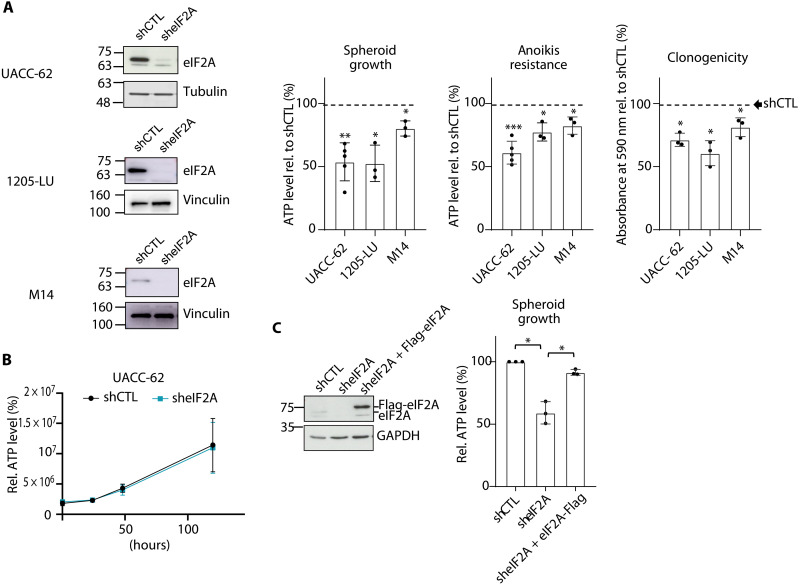
eIF2A promotes tumorigenic traits in metastatic melanoma cells. (**A**) Effect of eIF2A depletion on spheroid formation, anoikis resistance, and clonogenicity in various melanoma cell lines. Depletion efficiency (shRNA #7) was assessed by Western blot (left). Bar graphs depict the mean ± SD of three to five independent biological replicates, each with at least five technical replicates. Data are represented relative to their respective shCTL (dashed line). (**B**) Depletion of eIF2A does not cause defects in proliferation. Viable UACC-62 cells were measured with CellTiter-Glo. Graphic depicts the mean ± SD of four independent experiments. (**C**) Re-expression of eIF2A restores spheroid growth. sheIF2A cells were infected with a vector expressing sh-resistant Flag-tagged eIF2A or with empty vector. Expression of eIF2A was induced by the addition of doxycycline (DOX) (0.1 μg/ml) for 24 hours and assessed by Western blot with α-eIF2A antibody (left). Graphic depicts the mean ± SD of three independent biological replicates, each with at least five technical replicates. Statistics were calculated using two-tailed Student’s *t* test (*P < 0.05; **P < 0.01; ***P < 0.001). ATP, adenosine 5′-triphosphate.

To obtain insights into the role of eIF2A in melanoma progression, we tested the effect of eIF2A depletion on the tumorigenic properties of a panel of melanoma cell lines. We used stable expression of short hairpin RNAs (shRNAs) in metastatic UACC-62, 1205-LU, and M14 cells and assessed spheroid formation, anoikis resistance, and clonogenicity. All three metastatic traits were compromised after eIF2A down-regulation in all cell lines (Fig. 1A and fig. S1A). This occurred independently of cell proliferation, as two-dimensional (2D) growth was not affected by eIF2A down-regulation (Fig. 1B). Different shRNAs against eIF2A (sheIF2A) yielded similar results, indicating that the effects of eIF2A depletion are unlikely to result from off-target effects (fig. S1B). The effects were specific, as restoring eIF2A expression with shRNA-resistant Flag-tagged eIF2A rescued the phenotypic trait (Fig. 1C).

**Fig. 2.**
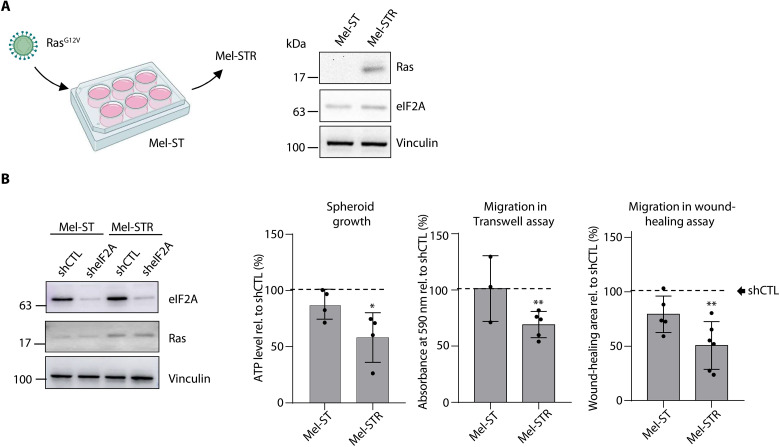
Acquired dependency on eIF2A upon *Ras* transformation. (**A**) Scheme to demonstrate the generation of Mel-STR cells from Mel-ST, and Western blot showing that eIF2A levels do not change upon *Ras* transformation. (**B**) Depletion of eIF2A affects traits in Mel-STR but not Mel-ST cells. Spheroid growth and migration were assessed after eIF2A knockdown (see representative Western blot to assess the efficiency of depletion on the left). Bar graphs depict the mean ± SD of three to five independent experiments and, in the case of spheroid, each consisting of five technical replicates. Data are represented relative to the respective shCTL (dashed line). Statistics were calculated using the two-tailed Student’s *t* test (*P < 0.05; **P < 0.01).

To further dissect the role of eIF2A in promoting metastatic traits, we aimed to compare its function in nontumoral versus tumoral settings. To avoid confounding effects from melanoma cell heterogeneity, we took advantage of a previously established system consisting of the immortalized melanocytic cell line Mel-ST and its metastatic counterpart, which is derived from Mel-ST upon H-Ras^G12V^ overexpression, named Mel-STR (Mel-ST + Ras) (*24*). Notably, eIF2A levels were not altered upon *H-Ras* transformation (Fig. 2A). We selected assays supported by both Mel-ST and Mel-STR cells, such as spheroid growth and migration, with the latter measured using Transwell and wound-healing assays. We found that depletion of eIF2A did not significantly affect the behavior of Mel-ST cells, whereas Mel-STR cells showed reduced growth and migration (Fig. 2B and fig. S2A). As observed with other metastatic cell lines, depletion of eIF2A did not affect proliferation of either Mel-ST or Mel-STR cells (fig. S2B). The requirement of eIF2A for migration was confirmed in metastatic UACC-62 cells (fig. S2C). Together, these results indicate that eIF2A promotes tumoral traits in metastatic melanoma cells independently of proliferation and that dependency on eIF2A is acquired upon oncogenic transformation.

### eIF2A does not overtly affect translation

**Fig. 3.**
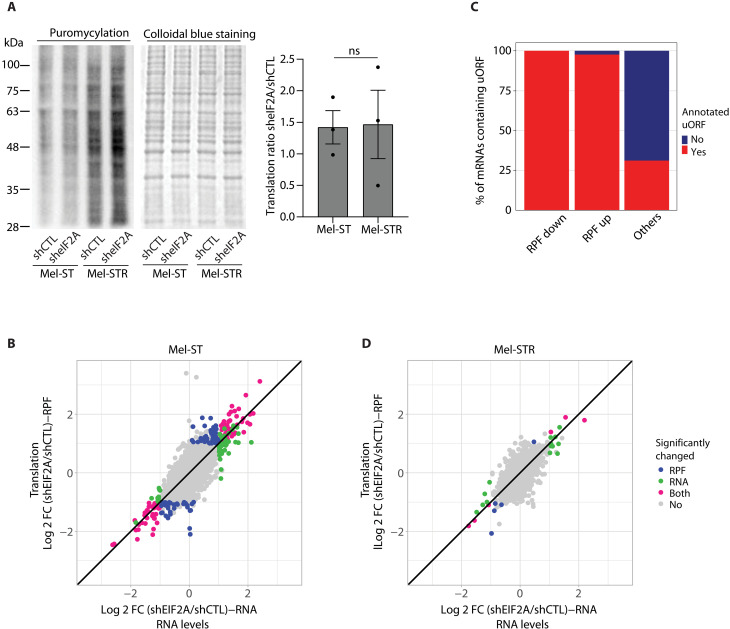
eIF2A does not overtly regulate translation in Mel-STR cells. (**A**) Effect of eIF2A depletion on global translation assessed by puromycylation. Western blot with α-puromycin antibodies (left) and colloidal blue loading control (middle). Translation was determined as the signal intensity of puromycin divided by global protein levels. Bar graph (right) shows the mean ± SD of the ratio of translation in sheIF2A versus shCTL cells (*n* = 3); ns, nonsignificant. (**B**) Ribosome profiling of Mel-ST cells. The scatter plot shows differences in RFPs between sheIF2A and shCTL cells against differences in RNA levels measured by RNA-seq. Each dot represents one gene, and colors indicate no significant change (gray), significant change at the RPF level (blue), significant change at the RNA level (green), or significant change at both the RPF and RNA level (pink) [*P* ≤ 0.05; log 2 fold change (Log 2 FC) ≥ 1]. Three independent biological replicates were carried for each condition. (**C**) Bar plot showing the proportion of mRNAs containing uORFs in the indicated study groups. (**D**) As in (B) for Mel-STR cells.

To explore whether the dependency on eIF2A in the metastatic versus the nontumoral contexts could be related to a differential function of eIF2A in translation promoted by oncogenic stress, we first assessed global translation upon depletion of eIF2A in both cell lines using puromycylation assays. We observed no changes at a global level in either Mel-ST or Mel-STR cells (Fig. 3A). This result could have been expected, as eIF2A has been described to affect mRNA-specific translation. Therefore, to identify mRNAs whose translation is specifically affected by eIF2A depletion, we performed ribosome profiling sequencing (Ribo-seq) (*25*). Independent biological triplicates were used to generate RNA sequencing (RNA-seq) and Ribo-seq libraries from both cell lines under shCTL and sheIF2A conditions. Libraries were highly reproducible (*r* = 0.97 to 1 between replicates; fig. S3, A and B), and quality assessment showed that they were enriched for coding sequence (CDS) reads and ribosome footprints of 32 nucleotides (nt) with the expected 3-nt periodicity enriched in frame 0 (fig. S3, C to E). In Mel-ST cells, some mRNAs (*n* = 78) exhibited significant changes at the ribosome-protected fragment (RPF) level, indicating that eIF2A regulates translation of a specific mRNA set in this cell line (Fig. 3B and table S1). The vast majority of these mRNAs harbor uORFs, consistent with previous reports showing a preference of eIF2A in regulating uORF-containing mRNAs (Fig. 3C) (*3*, *5*). Unexpectedly, depletion of eIF2A from Mel-STR cells had minimal effects on translation, with merely five mRNAs (*MEF2C*, *MAPRE3*, *ST8SIA1*, *CXCL8*, and *DENND4B*) in the altered “RPF only” group and six additional mRNAs (EIF2A, *ZNHIT1*, *RNF38*, *ANKRD1*, *ACTC1*, and *NPTX1*) showing concordant changes in RNA and RPF levels (Fig. 3D and table S1). These results indicate that eIF2A does not overtly regulate translation in the Mel-ST/Mel-STR context and point toward a translation-independent function of eIF2A upon metastatic transformation.

### eIF2A binds mRNAs in a translation-independent fashion

**Fig. 4.**
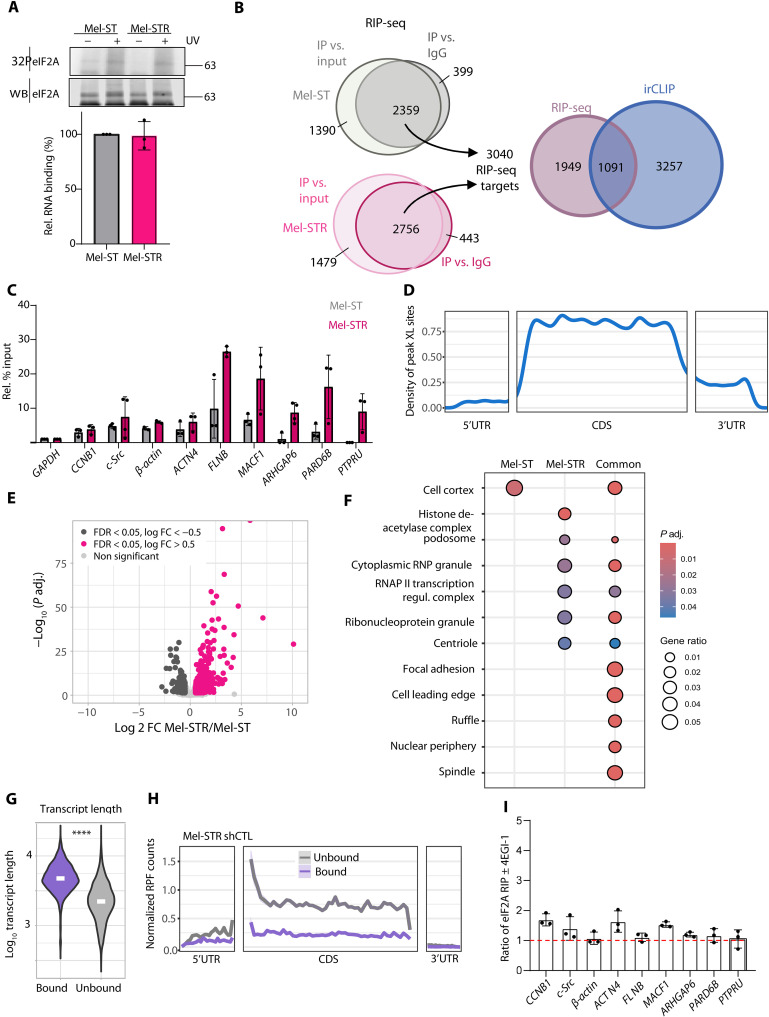
eIF2A binds mRNAs in a translation-independent fashion. (**A**) Global RNA binding proficiency of eIF2A in Mel-ST and Mel-STR cells. Top: PNK assays showing eIF2A-bound RNA labeled with ^32^P, and eIF2A protein detected by Western blot (WB). Bottom: Quantification of ^32^P signal corrected by immunoprecipitated eIF2A (*n* = 3). UV, ultraviolet. (**B**) Left: Venn diagrams depicting RIP-seq target selection in Mel-ST and Mel-STR cells. Transcripts were considered eIF2A targets when present in the eIF2A IP compared to both input and IgG control (*P* ≤ 0.05; Log 2 FC ≥ 1) in at least one cell line (*n* = 3). Right: Overlap of RIP-seq targets with infrared cross-linking and immunoprecipitation (irCLIP) targets identified in SK-Mel-147 cells. irCLIP targets were considered when peaks were detected in at least two replicates. (**C**) Validation of RIP-seq results by RIP–RT-qPCR in Mel-ST (gray) and Mel-STR (pink) cells. Bar graphs depict mean ± SD relative to input and normalized to *GAPDH* mRNA (*n* = 3 to 4). (**D**) Metagene analysis depicting the density of eIF2A irCLIP peaks along CDS and untranslated regions (UTRs) for targets identified both in irCLIP sequencing and RIP-seq. (**E**) Volcano plot showing differentially enriched eIF2A targets. (**F**) Gene ontology (GO) analysis of eIF2A targets. (**G**) Violin plot comparing the mRNA length of eIF2A targets and nontargets in Mel-ST and Mel-STR cells. mRNA length was calculated based on the most expressed isoform. White line represents the mean. Statistics were calculated using the Wilcoxon test (****P < 2.2 × 10^–16^). (**H**) Metagene analysis of normalized RPF counts in Mel-STR cells for eIF2A targets (blue) versus nontargets (gray). Footprint reads were normalized with differential expression sequencing 2 (DESeq2) size factor. (**I**) RIP–RT-qPCR of eIF2A targets in Mel-STR cells in the presence or absence of 4EGI-1. The ratio of eIF2A-bound mRNA ± 4EGI-1 was calculated and normalized to *GAPDH* mRNA. Bar graphs show mean ± SD of three independent biological replicates. Dashed line shows no difference. Statistics were calculated using the two-tailed Student’s *t* test.

The fact that eIF2A does not regulate translation in Mel-STR cells was unexpected and raised the question of how eIF2A promotes tumoral traits. As eIF2A is an RNA-binding protein, we first tested whether the RNA- binding activity of eIF2A was altered in Mel-STR compared to Mel-ST cells. Using T4 polynucleotide kinase (PNK) assays (see Materials and Methods), we measured global RNA-binding activity and found that eIF2A binds RNA similarly in both cell lines (Fig. 4A). We next searched for potential differences in the specific mRNAs being bound in the two cellular contexts using RNA immunoprecipitation followed by sequencing (RIP-seq). Quality control showed a high correlation between three independent biological replicates (*r* = 0.99 to 1; fig. S4, A and B). We performed enrichment analysis versus immunoglobulin G (IgG) controls and input samples carried in parallel and considered a target positive when it was enriched in the eIF2A immunoprecipitation (IP) compared to both IgG and input (log 2 fold change > 1; adjusted *P* < 0.05). Together, we found 3040 mRNAs bound by eIF2A using these thresholds (Fig. 4B and table S2). We validated a subset of these targets, including *CCNB1* and *c-Src*, which have been already described to bind eIF2A (*8*, *19*), using RIP coupled to reverse transcription quantitative polymerase chain reaction (RT-qPCR) and confirmed their enrichment over input relative to *glyceraldehyde-3-phosphate dehydrogenase* (*GAPDH*) mRNA (Fig. 4C).

We also performed infrared cross-linking and immunoprecipitation (irCLIP) of eIF2A in metastatic SK-Mel-147 cells, to strengthen our RIP-seq results and search for targets directly bound by eIF2A. The data show that about one-third of RIP-seq targets (*n* = 1091; 35.8%) are directly bound by eIF2A (Fig. 4B and table S4). In contrast to previously published data (*26*), our irCLIP results show that eIF2A binding to mRNAs is strongly enriched compared to the noncross-linked samples (34 versus 4%), whereas only 22% of all reads belong to the 18*S* ribosomal RNA (compared to 18% in the noncross-linked samples) (fig. S4C). The majority of eIF2A irCLIP peaks in mRNAs fall along the CDS (Fig. 4D), further suggesting a function of eIF2A independent of its reported role in translation initiation (Fig. 4D).

We next performed differential expression analysis of the RIP-seq data using DESeq2 to test whether eIF2A targets (whether direct or indirect) were differentially bound in the nontumoral versus the metastatic background. The large majority of targets (*n* = 2257) were bound similarly in both cell lines, while 237 and 546 targets were preferentially bound in Mel-ST and Mel-STR, respectively (Fig. 4E). Gene ontology (GO) analysis showed enrichment for terms related to cellular structures involved in cell adhesion and migration, pointing toward a function of eIF2A in migration via its mRNA binding function (Fig. 4F and table S3).

We next overlapped the Ribo-seq, RNA-seq, and RIP-seq data to assess direct regulation of translation or RNA levels by eIF2A (fig. S4, D and E). Only few mRNAs bound by eIF2A (51 of 3040) were regulated at the mRNA or translation level in either Mel-ST or Mel-STR cells, indicating that observed changes are mostly indirect. eIF2A mRNA targets are significantly longer than nontarget mRNAs, mainly due to longer CDSs and 3′ untranslated regions (3′UTRs) (Fig. 4G and fig. S4F). In addition, eIF2A mRNA targets are poorly translated compared to nontargets in both cell lines whether or not eIF2A is present, indicating that poor translation is an inherent feature of this mRNA group (Fig. 4H and fig. S4G).

The results so far imply that eIF2A binds mRNA in a translation-independent fashion. To support this hypothesis, we inhibited translation using inhibitors of initiation (4EGI-1; fig. S4H) or elongation (puromycin) and compared eIF2A binding to a subset of targets. We found that binding of eIF2A does not decrease in the presence of translation inhibitors (Fig. 4I and fig. S4I). This is especially relevant in the case of 4EGI-1, because this inhibitor acts by blocking the assembly of the cap-binding complex, a step before eIF2A function in initiation codon recognition during translation (*27*). These data indicate that the ribosome does not mediate the binding of eIF2A to its mRNA targets. Together with the poor translatability of eIF2A targets, these results strongly suggest that eIF2A binds mRNAs in a translation-independent fashion.

### eIF2A enforces centrosome composition and controls the directionality of migration

**Fig. 5.**
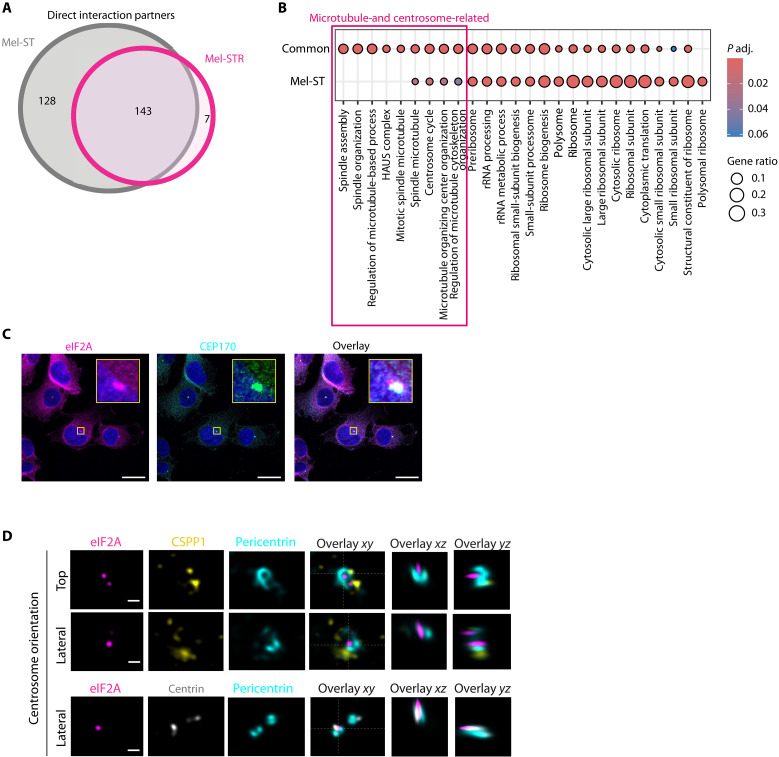
eIF2A localizes to the centrosome. (**A**) Identification of the eIF2A interactome. Venn diagram showing RNA-independent (i.e., direct) protein interactors of eIF2A in Mel-ST and Mel-STR cells, identified using SAINT (*n* = 3; BFDR ≤ 0.5). (**B**) GO term analysis of interactors found significant only in Mel-ST or in both Mel-ST and Mel-STR cells (“common”). GO terms related to microtubules and the centrosome are highlighted. rRNA, ribosomal RNA. (**C**) Confocal imaging of eIF2A and the centrosomal protein CEP170. Enlargements of the centrosomal region are shown in the inset. Scale bars, 20 μm. (**D**) eIF2A signal overlaps with the centriole. Super-resolution Airyscan microscopy images showing eIF2A (pink), CSPP1 (yellow), PCNT (turquoise), and centrin 3 (gray). Orthogonal *xz* and *yz* views are provided on the right. Scale bars, 500 nm (*n* = 2).

To understand the function of eIF2A in metastatic cells, we identified its protein interactome. We performed large-scale eIF2A IP from both Mel-ST and Mel-STR cells and identified associated proteins by liquid chromatography–tandem mass spectrometry (LC-MS/MS). Three independent biological replicates were performed using IgG as negative control, with or without ribonuclease I (RNase I), to distinguish RNA-independent interactions. Comparison of spectral counts showed a good correlation among replicates (*r* = 0.98 to 1) (fig. S5, A and B). We identified 354 interactors in Mel-ST and 230 interactors in Mel-STR using Significance Analysis of INTeractome (SAINT) and a Bayesian false discovery rate (BFDR) ≤ 0.05 (table S5). From these, 271 and 150 were RNA-independent (hereafter termed “direct”) interactors, respectively, most of which were shared between the two cell lines (Fig. 5A and table S5). Notably, the subset of significant interactions only in Mel-ST cells was related to translation, consistent with the stronger role of eIF2A in translation in this cell line (Fig. 5B). GO terms related to microtubule organization and centrosomal function appeared in the common group (Fig. 5B and table S6). Centrosomes are the primary microtubule-organizing center, and several of the eIF2A direct protein interactors are key centrosomal components [e.g., CEP170, CEP350, pericentrin (PCNT), pericentriolar material 1 (PCM1), NEDD1, AKAP9, and CDK5RAP2; see tables S5 and S6). Coimmunofluorescence of eIF2A with the centrosomal protein CEP170 revealed that eIF2A colocalizes with the centrosome in intact cells (Fig. 5C and movie S1). This was confirmed by costaining with another centrosomal protein, PCNT, in Mel-STR and UACC-62 cells (fig. S5C). Localization of eIF2A to the centrosome did not depend on microtubule dynamics, as it was not affected by cold-mediated microtubule depolymerization (fig. S5D).

The centrosome consists of the centrioles and the PCM and is surrounded by centriolar satellites, which facilitate protein trafficking and centrosome function (*28*). To gain granularity into the localization of eIF2A, we performed super-resolution Airyscan imaging with markers of these compartments, namely, centrin, PCNT, and CSPP1. Coimmunofluorescence of eIF2A with PCNT and CSPP1 shows that eIF2A is found inside the PCNT ring, which is known to surround the centriole (Fig. 5D, top and middle). Coimmunofluorescence of the centriolar marker centrin and eIF2A shows overlapping signals, indicating that eIF2A is found within centrioles (Fig. 5D, bottom).

**Fig. 6.**
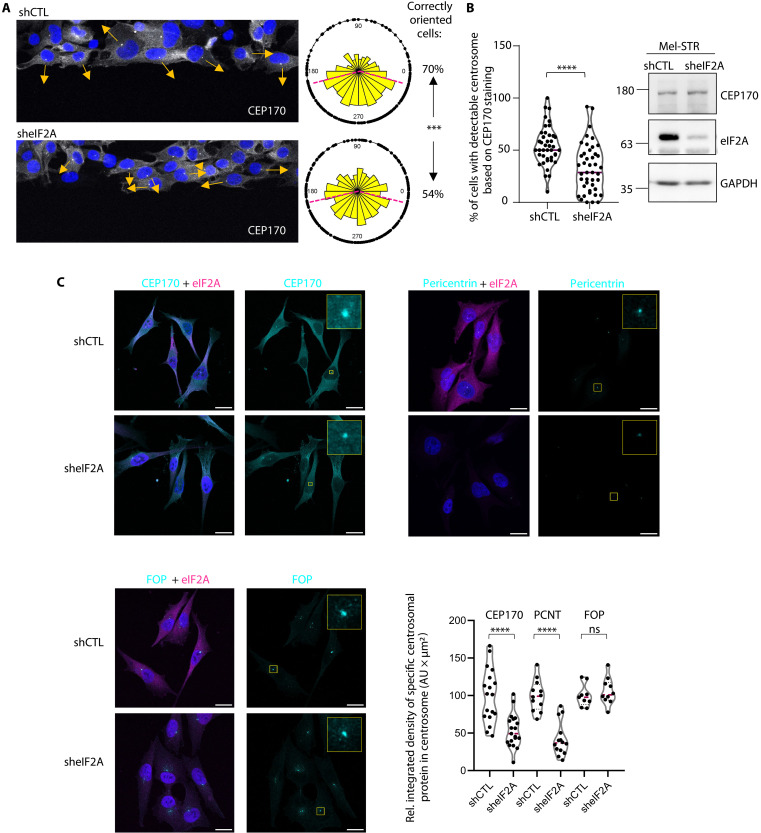
eIF2A functions in centrosome orientation and maintenance. (**A**) eIF2A promotes centrosome orientation during migration. Assessment of centrosome position in Mel-STR cells next to a wound 30 min after wound generation. Arrows indicate the directionality of migration based on the orientation of the centrosome with respect to the nucleus. Centrosomes located in a 150° circular sector facing the direction of wound closure were considered properly located. Pie charts show the localization of centrosomes in shCTL (*n* = 230) and sheIF2A (*n* = 309) cells. The plane was divided into 15° circular sectors with the nucleus at the center. The radius of each sector represents the relative number of cells with centrosomes located in that sector. Results are based on three independent experiments. A two-sample test for equality of proportions with continuity correction was applied to assess whether the difference in the distribution is significant (***P = 0.000124). (**B**) Percentage of cells next to the wound with detectable CEP170 signal. Overlap with the Golgi marker GM130 was used to confirm centrosome identity. Left: Violin plots with median (pink line), where each dot corresponds to the mean integrated density of 7 to 15 cells (*n* = 3). Statistics were calculated using unpaired two-tailed Student’s *t* test (****P < 0.0001). Right: Global CEP170 levels assessed by Western blot. (**C**) Coimmunofluorescence of eIF2A (magenta) and different centrosomal proteins (cyan) in UACC-62 shCTL and sheIF2A cells. Insets show magnification of the centrosomal region. Scale bars, 20 μm. Violin plots depict the median of integrated densities at the centrosome. Each dot represents the mean integrated density of the indicated centrosomal protein in the centrosomes of 5 to 20 cells (*n* = 3). Statistics were calculated using unpaired two-tailed Student’s *t* test (****P < 0.0001). AU, arbitrary units.

The results above raise the possibility that eIF2A participates in centrosomal function. Centrosomes are major microtubule-organizing centers (*29*). Given the requirement of eIF2A for directed cell migration, a process that depends on microtubule dynamics, we wondered whether eIF2A affected microtubule remodeling. We used fluorescence lifetime imaging microscopy (FLIM) of SiR-tubulin, a live-cell fluorescent probe that binds microtubules (*30*), to measure microtubule stability upon depletion of eIF2A from UACC-62 cells. The results show no differences in the SiR-tubulin modulation lifetime or in the phasor plot in sheIF2A versus shCTL (fig. S6A). In addition, microtubule regrowth assays involving cold-induced depolymerization followed by regrowth at 37°C showed efficient nucleation by the centrosome in sheIF2A cells (fig. S6B). These results suggest that microtubule dynamics are unaffected by sheIF2A depletion. However, centrosomes are also crucial for establishing cell polarity, which is essential for directional cell migration [reviewed in (*31*, *32*)]. In this process, centrosome orientation toward the direction of migration is key. Thus, we assessed whether eIF2A was involved in centrosome positioning by depleting eIF2A and staining for CEP170 and the cell nucleus in wound-healing assays 30 min after wound generation. We considered centrosomes located in a 150° circular sector facing toward the direction of wound closure as properly located. We observed a significant difference upon eIF2A depletion, as 70% of shCTL cells contain a correctly positioned centrosome while this percentage dropped to 54% in the case of sheIF2A cells (Fig. 6A). These results indicate that eIF2A promotes centrosome positioning and appropriate directionality of migration.

We noticed that it was more difficult to detect centrosomes in sheIF2A cells compared to shCTL cells based on CEP170 staining. We used automated image analysis to quantify the percentage of cells with detectable centrosomes, confirming this observation (Fig. 6B, left). This occurred in the absence of any defect on global CEP170 levels (Fig. 6B, right), suggesting that eIF2A is involved in recruiting or maintaining CEP170 in the centrosome. To further corroborate and extend these results, we measured centrosomal CEP170 intensity in UACC-62 cells and included PCNT and FGFR1 oncogene partner (FOP), another centrosomal protein (Fig. 6C). eIF2A depletion significantly reduced CEP170 and PCNT intensities, whereas FOP was not affected. Global CEP170 and PCNT levels were not reduced as assessed by Western blot (fig. S6C). We further confirmed these results by performing PCNT and FOP costaining and quantifying the levels of PCNT relative to FOP (fig. S6D). These data imply that eIF2A promotes centrosome composition by stabilizing the presence of specific components. Notably, changes in centrosome composition are not explained by variations in centriole number, which remain constant upon eIF2A depletion (fig. S6E).

### eIF2A promotes migration in an RNA-dependent but translation-independent fashion

**Fig. 7.**
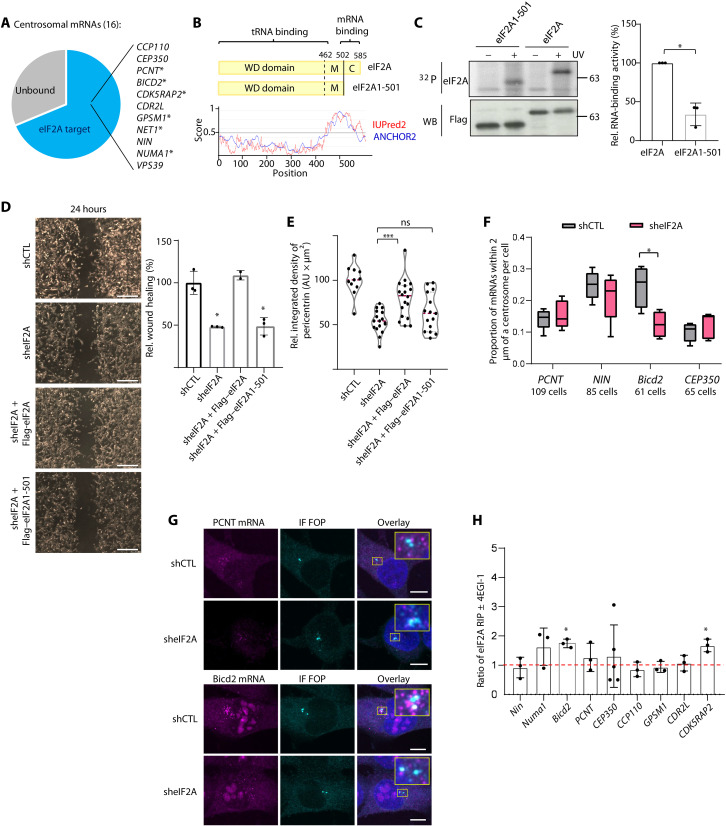
eIF2A promotes migration in a translation-independent fashion. (**A**) Reported centrosome-localized mRNAs that are eIF2A RIP-seq targets. Asterisks indicate direct targets detected by irCLIP. (**B**) eIF2A domains and disorder scores based on IUPred2A and ANCHOR2. (**C**) PNK assay to assess the RNA binding efficiency of proteins depicted in (B). Bottom: Quantification of the ^32^P signal corrected by the amount of immunoprecipitated protein (*n* = 3). (**D**) eIF2A mRNA binding is required for migration. Mel-STR sheIF2A cells were transfected with plasmids expressing sh-resistant Flag-tagged eIF2A or its C-terminal deletion derivative and tested in wound-healing assays. Representative images are shown at the left. Scale bars, 500 μm. Bar graphs depict mean ± SD relative to shCTL (*n* = 3). (**E**) eIF2A mRNA binding enforces centrosome composition. Violin plots depict the median of integrated density of PCNT at the centrosome. Every dot represents the mean integrated density of 5 to 15 cells (*n* = 4). (**F**) Effect of eIF2A depletion on mRNA accumulation at the centrosome, assessed by single molecule fluorescence in situ hybridization (smFISH). Box plots depict the proportion of the indicated mRNAs within 2 μm of a centrosome. Horizontal bars indicate the mean. Significance was assessed with one-sided Welch’s *t* test. (**G**) Representative micrographs of UACC-62 shCTL and sheIF2A cells used for quantification in (F), imaged by wide-field microscopy. Magnifications of the centrosome region are shown in the insets. Scale bars, 10 μm. (**H**) eIF2A binds centrosomal mRNAs in a translation-independent fashion. RIP-qPCR of centrosomal mRNA targets in the presence or absence of 4EGI-1. The ratio of eIF2A-bound mRNA ± 4EGI-1 was calculated and normalized to GAPDH mRNA. Bar graphs show mean ± SD (*n* = 3). Dashed line shows no difference. Unless otherwise indicated, statistics were assessed using paired (C, D, and H) or unpaired (E) two-tailed Student’s *t* test (*P < 0.05; ***P < 0.001).

The PCM is an amorphous matrix consisting of proteins and specific “centrosomal” mRNAs whose enrichment at the centrosome is regulated (*33*–*37*). Given the localization of eIF2A to the centrosome and its function in RNA binding, we explored whether mRNAs known to localize to the centrosome are bound by eIF2A. Our RIP-seq data show that the large majority of reported centrosome-localized mRNAs (69%) are eIF2A targets, half of which (54%) bind directly to eIF2A (Fig. 7A and table S2). Some of the eIF2A targets (GPSM1, PCNT, CEP350, and CCP110) show increased binding to eIF2A in Mel-STR cells. The high frequency of binding to centrosomal mRNAs suggested the possibility that eIF2A regulates the localization or accumulation of these mRNAs to the centrosome, thus impacting centrosomal function. To address this possibility, we first tested whether the RNA binding activity of eIF2A was important for migration. eIF2A consists of a tryptophan-aspartic acid (WD)-repeat domain that folds into a β-propeller structure responsible for tRNA binding, a middle domain (M) thought to interact with eIF5B, and a C-terminal region (C) required for mRNA binding (Fig. 7B) (*6*, *38*). Both the M and C domains are highly disordered (Fig. 7B). We generated a C-terminally truncated version of eIF2A (eIF2A1-501) and corroborated that this mutant exhibits reduced RNA-binding activity using PNK assays (Fig. 7, B and C). We then tested the capacity of this mutant to restore eIF2A function in migration in cells that had been depleted of eIF2A. Wound-healing assays showed that the migration phenotype caused by eIF2A depletion was fully rescued by overexpression of sh-resistant, full-length Flag-eIF2A, but not by C-terminally truncated eIF2A (Fig. 7D). Consistent with these results, full-length eIF2A, but not the C-terminally truncated mutant, could rescue PCNT levels at the centrosome (Fig. 7E and fig. S7). These data indicate that eIF2A RNA binding is required to enforce centrosome composition and orientation for migration.

We next tested whether eIF2A influenced the localization of mRNAs at the centrosome using single molecule fluorescence in situ hybridization (smFISH). Of the four tested centrosome-enriched transcripts that bind eIF2A, only one showed decreased accumulation at the centrosome upon eIF2A depletion (Fig. 7, F and G), suggesting no major role of eIF2A in centrosomal mRNA localization. As the recruitment of mRNAs to the centrosome is thought to occur cotranslationally (*33*, *35*, *36*, *39*, *40*) [reviewed in (*34*)], these data also imply a minor role of eIF2A in local translation. In addition, binding of eIF2A to centrosomal transcripts was insensitive to the inhibitor 4EGI-I, indicating that eIF2A binds to centrosomal mRNAs in a translation-independent fashion (Fig. 7H). These results indicate that eIF2A promotes migration independently of translation.

## DISCUSSION

Migration plays a crucial role in cancer progression, particularly in the process of metastasis where cancer cells move from the primary site to establish secondary tumors in distant organs (*41*, *42*). Migration involves complex interactions between different cellular structures, including microtubules and the centrosome (*43*, *44*). Microtubules facilitate the dynamic reorganization of the cytoskeleton, enabling cells to polarize, change shape, and move. Centrosomes are the main microtubule-organizing centers and, hence, function at the nexus of these critical processes (*29*). Here, we uncover a function of the alternative translation initiation factor eIF2A in promoting migration by enforcing centrosome composition and orientation. Unexpectedly, although this function is based on the RNA-binding activity of eIF2A, it is independent of translation.

eIF2A colocalizes with the centrosome and interacts with numerous proteins of the PCM (CDK5RAP2, PCNT, TUBG1, TUBGCP2, and TUBGCP3) as well as the centrosome subdistal appendages (CEP170 and NIN). Both structures play a key role in anchoring and stabilizing microtubules, making them essential for cell division, intracellular transport, and maintaining cell shape and polarity, which are critical for migration (*45*). eIF2A also interacts with a large fraction of centrosome-localized mRNAs. Some of these interactions are detected preferentially in Mel-STR cells (for example, interactions with PCNT, CCP110, and CEP350 mRNAs), but, notably, they are independent of translation. What is, then, this translation-independent function of eIF2A? One possibility is that eIF2A is directly involved in the localization of centrosome-enriched mRNAs by interaction with motor proteins such as DYNLL1, responsible for PCNT mRNA transport to the centrosome (*36*). However, we find that accumulation of most of the tested mRNAs (including PCNT) to the centrosome is not affected by eIF2A depletion. Another possibility is that eIF2A performs a stabilizing function at the centrosome. Recent results suggest that the mitotic centrosome is organized following principles of molecular condensation (*46*–*48*). Condensation is stimulated by multivalent protein-protein and protein-RNA interactions, facilitated by intrinsically disordered regions in proteins (*49*). Notably, it is the disordered region of eIF2A that is required for cell migration. Furthermore, eIF2A binds to long and poorly translated mRNAs in addition to a large number of PCM proteins, and the accumulation of some of them at the centrosome decreases upon eIF2A depletion, whereas their global levels remain unchanged. Thus, eIF2A could perform a scaffolding function, promoting protein-protein and protein-RNA interactions at the PCM, ultimately resulting in centrosome stabilization. It would be interesting to decipher whether this stabilization follows the principles of biomolecular condensation in interphase centrosomes. Alternatively, eIF2A could be involved in microtubule anchoring or actin-dependent movement of the centrosome, two processes that impact centrosome positioning and directed migration (*45*).

eIF2A acquires a function in migration only after oncogenic transformation. The mechanism by which this function emerges in metastatic cells is unclear, but it is tempting to speculate that local modification of eIF2A at the centrosome may contribute to this role. In nontumoral Mel-ST cells, eIF2A is not only unnecessary for cell migration but also plays a more prominent role in translation regulation, in agreement with stronger interactions with the translational machinery. Most translationally regulated transcripts contain uORFs, consistent with previous reports in other systems (*3*, *5*). However, most uORF-mediated regulation appears to be indirect, because the majority of regulated mRNAs do not bind (either directly or indirectly) to eIF2A. This might be a broader phenomenon, as information on eIF2A binding to translationally regulated transcripts in previous reports is lacking. Identifying the mediator(s) through which eIF2A influences translation of uORF-containing transcripts will be an important direction of future research.

Last, although we focus on centrosome interactions in this work, there are other interactions of eIF2A identified in this study that could contribute to tumor progression. Ribosome biogenesis, for example, is a process frequently altered in cancer and also a prominent functional term of the eIF2A interactome.

In summary, our findings uncover a role of eIF2A in promoting centrosome composition and migration without overtly regulating translation. These findings challenge the traditional view of eIF2A as solely a translation initiation factor and open avenues for understanding the complex mechanisms contributing to melanoma metastasis.

### Limitations of the study

Our experiments with the translation inhibitor 4EGI-1 indicate that eIF2A binds to its targets for a purpose unrelated to translation. However, although centrosomal mRNAs are not listed in the IRES database (*50*), we cannot fully exclude the possibility that they initiate translation via a noncanonical, cap-independent mechanism, in which case, 4EGI-1 would be ineffective. In addition, while our global ribosome profiling may lack the resolution to detect localized translation changes at the centrosome, the fact that eIF2A binds to poorly translated mRNAs and that this binding occurs independently of translation supports a more direct role of eIF2A in the centrosome. Further research is needed to clarify the molecular mechanisms by which eIF2A regulates centrosome composition and orientation. Studying the role of eIF2A in the migration of other cancer types could reveal broader implications for targeting eIF2A therapeutically.

## MATERIALS AND METHODS

### Cell culture

Melanoma cells (UACC-62, 1205-LU, M14, and SK-Mel-147) and other cell lines [human embryonic kidney (HEK) 293 T, Phoenix] were cultured in Dulbecco’s modified Eagle’s medium (DMEM)–GlutaMAX (Gibco, 31966021) supplemented with 10% fetal bovine serum (FBS) (Gibco, 10270106) and 1% penicillin-streptomycin (Gibco, 15070063) at 37°C and 5% CO_2_. Mel-ST and Mel-STR cells were cultured under similar conditions with 7% FBS. Cells were regularly tested for mycoplasma infection and were authenticated using the GenePrint 10 System in the Genomics Unit of the Spanish National Cancer Research Center (Madrid).

### Cloning and plasmids

SMARTvector lentiviral constructs expressing short hairpins targeting eIF2A (V3SVHS07_4951697, V3SVHS07_7121150, and V3SVHS07_9747158) or a nontargeting hairpin (77VSC11715) were obtained from Horizon Discovery. pCDNA3.1 constructs expressing Flag-eIF2A and Flag–eIF2A1-501 were provided by S. K. Yang (*6*). Point mutations not altering the eIF2A amino acid sequence were introduced in the targeting region of sheIF2A V3SVHS07_9747158 to generate Flag-eIF2A or Flag–eIF2A1-501 resistant to the shRNA for rescue experiments. Mutations were as follows: eIF2A wild type: TGCCTTGAATTCTCACCCAAA to sh-resistant: TGTCTAGAGTTTTCTCCGAAA (Cys^79^-Lys^85^). The sh-resistant version of Flag-eIF2A was also introduced into pCW57.1 to express Flag-eIF2A upon doxycycline induction.

### Viral infections

HEK-293 T cells were used for lentiviral shRNA and Flag-eIF2A expression, while Phoenix-AMPHO cells were used for retroviral H-Ras^G12V^ expression. Cells were transfected using the calcium phosphate method to produce viral particles as previously described (*23*).

### In vitro assays for tumoral traits

Spheroid formation, anoikis resistance, and clonogenicity assays were performed as previously described (*23*). Wound-healing assays were performed in a two-well culture insert (ibidi, #81176). Twelve thousand cells were seeded in each chamber and grown overnight for generation of a monolayer before the insert was removed. Two milliliters of cell culture medium were added, and pictures of the gap were taken at 0, 7, or 24 hours using the microscope Leica Inverted DMI6000B (5×/0.12 objective). Wound healing was quantified by measuring the percentage of filled space in the insert using a Fiji ImageJ plugin (*51*).

Transwell assays were performed using 8-μm-pore Transwell filters (Cultek, 153464). Fifty thousand cells were seeded in 200 μl of serum-free DMEM on top of each membrane, and 500 μl of DMEM with 10% serum was placed in the bottom chamber. After 15 hours, the insert was washed with phosphate-buffered saline (PBS), and cells on the lower surface of the membrane were fixed with methanol and stained with crystal violet. To quantify migration, we dissolved crystal violet in 10% acetic acid and measured the absorbance at 590 nm.

### Protein extract preparation and immunoblotting

Protein extraction and immunoblotting were performed as previously described (*23*). The following primary antibodies and dilutions were used for the Western blot: α-eIF2A (Abcam, #ab169528; 1:1000), α-Flag (Sigma-Aldrich, #F-3165; 1:1000), α-GAPDH (Abcam, #ab8245; 1:1000), α-tubulin (Sigma-Aldrich, #T9026; 1:5000), α-vinculin (Sigma-Aldrich, #V9131; 1:1000), α-puromycin [Developmental Studies Hybridoma Bank (DSHB), #PMY-2A4; 0.25 μg/ml], α-PCNT (Abcam, #ab220784; 1:1000), and α-CEP170 (Invitrogen #41-3200; 1:1000). Secondary antibodies were used at 1:1000 dilution: horseradish peroxidase– or fluorescence-coupled anti-rabbit or anti-mouse IgG (GE HealthCare, #NA934V and #NA931V; LICOR, #926-32210 and #925-68073).

### PNK assay

PNK assays were performed as previously described (*23*), whereas IP and washes were adapted to eIF2A. Specifically, eIF2A was immunoprecipitated using 40 μl of Dynabeads A previously coupled to 2.6 μg of eIF2A antibody (Abcam, #ab169528) for 2 hours at 4°C with rotation. The beads were then washed four times with 200 μl of high-salt washing buffer [500 mM NaCl, 10 mM tris (pH 7.4), 0.5 mM EDTA, 0.5% NP-40, and 1× protease inhibitor cocktail (PIC)] and twice with PNK buffer [50 mM tris, 50 mM NaCl, 10 mM MgCl_2_, 0.5% NP-40, and 5 mM dithiothreitol (DTT)].

### Puromycin labeling assay

Cells were grown until 70% confluence and incubated with puromycin (10 μg/ml) for 30 min at 37°C. As negative control, cells were incubated with cycloheximide (CHX; 100 μg/ml) for 5 min before adding the puromycin. Cells were harvested on ice by scraping, lysed in radioimmunoprecipitation assay buffer, and analyzed by Western blotting using an anti-puromycin antibody (DSHB, #PMY-2A4; 0.25 μg/ml). As a loading control, blue colloidal staining was performed according to the manufacturer’s instructions (Invitrogen, #LC6025).

### RNA immunoprecipitation–quantitative polymerase chain reaction

Cells derived from half 150-mm plate were lysed in hypotonic gentle lysis buffer (10 mM tris-HCl, 10 mM NaCl, 2 mM EDTA, and 0.5% Triton X-100) and centrifuged for 10 min at maximum speed at 4°C. Ten percent of the supernatant was taken for Western blot and RNA extraction as input samples. The remaining supernatant was diluted in 1× NET (50 mM Tris-HCl, 150 mM NaCl, 0.1% NP-40, and 1 mM EDTA) with RNase inhibitor (Promega, #N2111) and incubated for 2 hours at 4°C on a wheel with 50 μl of Dynabeads A previously coupled to 3 μg of either α-eIF2A (Abcam, #ab169528) or IgG antibody. The beads were then washed five times with 500 μl of 1× NET buffer. Ten percent of the beads were reserved for Western blot analysis, and RNA was extracted from the remaining beads using TRIzol. SuperScript II (Thermo Fisher Scientific, #1806414) was used to retrotranscribe, and the resulting cDNA was used for qPCR using SYBR Green Master Mix (Applied Biosystems, #4367659) with a ViiA 7 real-time PCR system with specific primers (sequences in table S7). Relative quantifications were calculated with the ΔΔCT method.

### RNA immunoprecipitation sequencing

RIPs were performed as described above for RIP-qPCR, except that RNA was extracted from the beads after proteinase K (Ambion, #AM2546) digestion for 30 min at 55°C before phenol-TRIzol extraction. Polyadenylate [poly(A)] selection and library preparation for Illumina sequencing were performed by the Centre for Genomic Regulation (CRG) Genomics Unit. Samples were sequenced to at least 35 million reads on Illumina NextSeq 2000 [paired end; 50 base pairs (bp)] and analyzed as described in computational methods.

### Ribo-seq and RNA-seq

Mel-ST shCTL and sheIF2A and Mel-STR shCTL and sheIF2A cells were plated on 150-mm plates and grown to 80% confluency. Cells were incubated for 2 min in DMEM containing CHX (0.1 mg/ml), washed with PBS + CHX, lysed in ice-cold lysis buffer [10 mM tris-HCl (pH 7.4), 10 mM MgCl_2_, 100 mM NaCl, 1% Triton X-100, 2 mM DTT, 0.25% deoxycholate, and CHX (100 μg/ml)], scraped off, and snap-frozen in liquid nitrogen. Lysates were thawed at 25°C and cleared for 5 min at 12,000*g*. Aliquots for Western blot and RNA-seq were taken at this step. For Ribo-seq, six units of absorbance at 260 nm per replicate were digested with 36 U of RNase I per unit (Ambion, #AM2294) for 10 min at 22°C under rotation, and digestion was stopped by adding RNAsin. Polysome profiling was performed as previously described (*52*). Monosome peaks were collected, and RNA was extracted. RPFs were purified via polyacrylamide gel electrophoresis on a 15% tris-borate EDTA–urea gel using PAGExt (Immagina, #KGE002), and library preparation from these fragments was prepared using LACEseq (Immagina, #LS-001) following the instructions of the manufacturer. Samples were sequenced with single-end 50-bp reads on NextSeq 2000 to at least 50 million reads per sample.

RNA-seq libraries were prepared from 1 μg of total RNA. Poly(A) selection and library preparation for Illumina sequencing were performed by the CRG Genomics Unit. Sequencing was performed with single-end 50-bp reads on NextSeq 2000 to at least 35 million reads. Ribo-seq and RNA-seq data were analyzed as described in the Supplementary Materials.

### Interactome capture and mass spectrometry

eIF2A IP was performed in Mel-ST and in Mel-STR cells (six replicates per cell type) as described for RIP-qPCR but using one 150-mm plate per IP. Half of the samples were digested with 100 U of RNase I (Ambion, #AM2294) for 15 min at 25°C and washed four more times with 1× NET buffer before on-bead enzymatic digestion and desalting. Specifically, beads were washed three times with 200 mM ammonium bicarbonate (ABC) and resuspended in 60 μl of 6 M urea in 200 mM ABC. Proteins were reduced by the addition of 3 μl of 10 mM DTT in 200 mM ABC and incubation for 1 hour at 37°C under shaking. Alkylation was achieved by adding 3 μl of 20 mM iodoacetamide in 200 mM ABC and incubating for 30 min at room temperature under shaking. Beads were diluted with 300 μl of 200 mM ABC and digested with trypsin (Promega, #V5111) through the addition of 5 μl of trypsin (0.2 μg/μl; overnight at 37°C, shaking). The supernatant was selected, acidified by adding 40 μl of 100% formaldehyde to achieve a pH of 2.5, and desalted using C18 UltraMicroSpin columns (The Nest Group, SUM SS18V9). Samples were analyzed by LC-MS/MS in an Orbitrap Lumos with a 60-min gradient by the CRG Proteomics Unit. Bovine serum albumin (BSA) was digested in parallel as a quality control and ran between each of the samples to avoid carryover and assess the instrument performance.

### Infrared cross-linking and immunoprecipitation

irCLIP was performed in SK-Mel-147 cells as previously described (*53*). irCLIP_ddRT primers used are listed in table S8. Quality of the libraries was confirmed by Bioanalyzer, and the libraries were sequenced using Illumina HiSeq 2500 (single read; 50 bp) at the CRG Genomics Unit. Data were processed as described in the “Computational analysis” section.

### Immunofluorescence

Cells were seeded on cover slides and fixed on the next day in 4% paraformaldehyde (PFA) for 15 min. Slides were incubated for 2 hours in blocking solution (PBS, 2% BSA, and 0.01% Triton X-100) containing the primary antibody [α-eIF2A–Alexa Fluor 647, 1:100 (Abcam, #ab215231); α-CEP170, 1:1000 (provided by I. Vernós’ laboratory); α-CEP170, 1:100 (Invitrogen, #41-3200); α-PCNT, 1:500 (Abcam, #ab270118); α-FOP, 1:1000 (Abnova, #H00011116-M01); α-CSPP1, 1:500 (Proteintech, #11931-1-AP); α-centrin 3 (Abnova, #H00001070-M01); α-centrin (Sigma-Aldrich, #04-1624); and α-GM130–Alexa Fluor 488, 1:20 (Abcam, #ab275987)] and washed three times with PBS containing 0.01% Triton X-100 and 0.5% BSA. Slides were then incubated for 1 hour in the dark with the corresponding fluorescent secondary antibodies (1:1000 in blocking solution), washed three times with PBS, and stained with 4′,6-diamidino-2-phenylindole (DAPI) (1 μg/ml) for 15 min. After two additional washes with PBS, slides were mounted in Fluoromount-G (SouthernBiotech) and stored at 4°C until analysis.

### Assessment of centrosome positioning

Twelve thousand Mel-STR cells were seeded in a two-well culture insert (ibidi, #81176) and grown overnight to a monolayer. The insert was then removed, and cells were incubated for 30 min before fixation with 4% PFA for 15 min. Immunofluorescence of CEP170 and GM130 was performed as described above, the nucleus was stained with DAPI, and cells were imaged using the Leica STELLARIS confocal microscope. The identity of centrosomes was corroborated by their proximity to the Golgi apparatus. To assess the centrosome orientation, we developed a custom pipeline in CellProfiler to automatically determine the centrosome position with respect to the nucleus and the leading edge. Specifically, images were preprocessed with a custom ImageJ macro, projecting each series of LIF file generated from the LAS X software in Z (standard deviation method) and splitting and converting all channels into independent TIF files. Subsequently, centrosome and nucleus coordinates were obtained for cells automatically classified as “outer” (next to the wound) or “inner” (not next to the wound). The data were analyzed with a custom implementation script in R using circular statistics (circular library) to calculate the centrosome orientation distribution and perform the Kuiper’s test of uniformity. All the scripts were assembled into a single workflow using Snakemake under the name “CellOrientation” and are accessible from https://doi.org/10.5281/zenodo.15125030.

### Microtubule nucleation assay

Microtubule regrowth assays were performed as described by Ezquerra *et al.* (*54*). Briefly, UACC-62 cells were grown overnight on cover glasses. To depolymerize microtubules, plates with cells are floated for 30 min on ice with water. Then, to induce microtubule regrowth, cover glasses were submerged for 10 or 20 s to the medium prewarmed to 37°C. Immediately after, cells were fixed in ice-cold methanol for 10 min on ice. Coimmunofluorescence of α-tubulin and a centrosomal marker was performed. Microtubule asters were quantified by generating a circular region of interest (ROI) of 5-μm diameter and placing it around the centrosome identified with the centrosome marker. Then, the intensities of the microtubule channel in the selected region were measured. Next, the ROI was moved to a nearby region with similar background intensity to determine the background intensity, which was subtracted from the measured aster intensity.

### Live-cell FLIM

To measure tubulin dynamics, we seeded 15,000 UACC-62 shCTL and sheIF2A cells in p8 glass-bottom plates (ibidi, #80807). The next day, cells were incubated with 500 nM SiR-tubulin (Spirochrome, #SC002) for 1 hour, and FLIM imaging was performed on Leica SP8 FALCON inverted confocal microscope equipped with white light laser (470 to 670 nm with an 80-MHz repetition rate) and an HC PL APO CS2 63×/1.4 oil-immersion objective using the LAS X software. Single-plane 512-by-512 images were acquired, with a voxel size of 0.1806 μm by 0.1806 μm by 1 μm (zoom factor of 2). The excitation wavelength was set to 645 nm, and photon emission was detected using a Leica hybrid photodetector for single molecule detection (HyD SMD) with five frame repetitions. Images representing photon counts per pixel and modulation lifetime (in nanoseconds) per pixel were exported from the SP8 FALCON system in PicoQuant file format (.ptu), and FLIM phasor analysis was performed using the FLIMPA open source software (*30*).

### Confocal microscopy

Images were acquired with the Leica STELLARIS 5 confocal microscope using the LAS X software equipped with a 405 diode laser, an optically pumped semiconductor laser 488, a diode-pumped solid-state laser 561, and a white light laser (485 to 685 nm) with an 80-MHz repetition rate, equipped with silicon-based hybrid detectors (HyD S) and using an HC PL APO CS2 63×/1.4 oil-immersion objective for colocalization studies and an HC PL APO CS2 40×/1.3 oil-immersion objective for centrosome orientation and quantification studies. 3D volume stacks were taken sequentially (in a frame-by-frame acquisition mode, 400-Hz, 1024 pixel–by–1024 pixel image format, system-optimized *z*-step size to achieve a voxel size of 0.2841 μm by 0.2841 μm by 0.3462 μm) using 405- (DAPI), 490- (Alexa Fluor 488), and 633-nm (Alexa Fluor 647) excitation lines with three independent HyDs. Fluorescence intensities of CEP170, PCNT, and FOP in the centrosome were measured using an ROI placed over the centrosome. To correct for differences in image acquisition, laser power, etc., we determined the background and substracted for each image specifically by measuring intensities inside the cells next to the centrosome.

Super-resolution Airyscan imaging was performed on a Zeiss LSM 980 Airyscan 2 microscope, controlled by ZEN Blue 3.2 software. Images were acquired using a C Plan-Apochromat 63×/1.4 oil-immersion objective and Zeiss 1.518 refractive index (Abbe number: 45) oil-immersion medium. For excitation, 488- (PCNT or centrin), 561- (CSPP1), and 639-nm (eIF2A) laser diodes were used. The voxel size was 44 nm by 44 nm by 100 nm (zoom factor of 6), and *z* stacks were acquired every 130 nm, covering the complete satellite volume. Raw 3D Airyscan images were preprocessed with the ZEN software Airyscan processing using the standard filter strength and 3D super-resolution (SR) processing adjusted per channel. Channel co-registration was performed using TetraSpeck fluorescent microspheres (200 nm; Invitrogen, #T14792) with the same imaging settings for channel alignment and chromatic aberration corrections for colocalization studies. Images were then processed with Huygens software (Scientific Volume Imaging), where images were first deconvolved using the measured point spread function obtained from the co-registered TetraSpeck beads (average full width at half maximum following deconvolution: *x* = 160 nm, *y* = 166 nm, *z* = 662 nm), and then, channels were aligned and corrected for chromatic aberration. Images were then visualized, and orthogonal views were obtained using Fiji/ImageJ (*55*).

### smFISH-immunofluorescence

To perform the smFISH, we generated probe sets of DNA oligonucleotides (GenScript) targeting mRNAs of interest based on the Oligostan script (see table S10) (*56*). The generation of transcript-specific primary RNA probe sets and smFISH-immunofluorescence were performed as previously described (*57*). smFISH imaging was performed on an Opera Phenix high-content screening system (Revvity), with a 63× water-immersion objective (numerical aperture of 1.15). 3D images were acquired, with a spacing of 0.6 μm.

To quantify mRNAs and evaluate the percentage localizing to the centrosome, we developed a pipeline named SmallFish, which is available at https://zenodo.org/records/15124658. It provides a ready-to-use graphical interface to combine Python packages for cell analysis. In the pipeline, cell segmentation (2D) was performed using cellpose (https://github.com/MouseLand/cellpose) (*58*); meanwhile, spot detection was carried out using BigFish (https://github.com/fish-quant/big-fish) (*59*). Spots were considered colocalizing when found within a radius of 2 μm of one another.

### Statistical analysis

All experiments have been carried out in at least three independent biological replicates. Experimental data are presented as means ± SD, and statistical analysis was carried out with GraphPad Prism 6 software unless otherwise indicated.

### Computational analysis

See the Supplementary Materials.

## Supplementary Materials

### The PDF file includes:

Supplementary Text Figs. S1 to S7 Legends for tables S1 to S6, S9, and S10 Tables S7 and S8 Legend for movie S1 References
