# Supplementary material for: eIF2A regulates cell migration in a translation-independent manner
Source: Sci Adv. 2025 Aug 1;11(31):eadu5668. doi: 10.1126/sciadv.adu5668 (PMC12315956; doi:10.1126/sciadv.adu5668)
Supplement: Supplementary file 1 — Supplementary Text Figs. S1 to S7 Legends for tables S1 to S6, S9, and S10 Tables S7 and S8 Legend for movie S1 References [file sciadv.adu5668_sm.pdf]

Science Advances
AAAS

Supplementary Materials for

eIF2A regulates cell migration in a translation-independent manner

Jennifer Jungfleisch *et al.*

Corresponding author: Fátima Gebauer, fatima.gebauer@crg.eu

Sci. Adv. **11**, eadu5668 (2025)
DOI: 10.1126/sciadv.adu5668

The PDF file includes:

Supplementary Text
Figs. S1 to S7
Legends for tables S1 to S6, S9, and S10
Tables S7 and S8
Legend for movie S1
References

Other Supplementary Material for this manuscript includes the following:

Tables S1 to S6, S9, and S10
Movie S1

SUPPLEMENTARY TEXT

Computational analysis

Ribo-Seq analysis

To process the Ribo-Seq data, reads were trimmed according to the indications from the manufacturer. First, the LACE-seq linker was trimmed from the 3' with cutadapt v4.1 (*60*). Then, the 5' and 3' unique molecular identifiers (UMIs) were extracted with the function 'extract' from UMI-tools (*61*) and finally, cutadapt was used again to cut the thymidine preceding the RPF. Reads from ribosomal RNA were removed with ribodetector (*62*) and the remaining reads were aligned to the genome hg38 reference with "spliced transcripts alignment to a reference" (STAR) (*63*) allowing a maximum of 2 mismatches and forcing end-to-end read alignment. Then, the function 'dedup' from UMI-tools (*61*) was run to remove duplicated sequences. For gene-centered analysis, read counts were obtained with featureCounts (*64*) taking only the reads aligned to the CDS (specified options -t CDS -g gene_id -O -J). In parallel, RNA-seq data was processed first by removing sequencing adaptors with TrimGalore (*65*), and then the reads were aligned to the genome hg38 reference with STAR(*63*) using ENCODE standard options. Read counts were obtained with featureCounts (*64*) using the same option as for RPFs.

Differential gene expression analysis was carried on with the Bioconductor R package DESeq2 (*66*), only genes with at least 10 mean normalized counts across the 6 samples under study were considered. For log2FoldChange analysis between sheIF2A and shCTL samples, DESeq was run considering either the counts from the RPFs or the total RNA grouping the samples according to shRNA conditions. Afterwards, the log2FolChange values between total RNA and RPFs were correlated. Genes with an absolute log2FoldChange higher than 1 and an adjusted p-value under 0.05 were considered regulated. This analysis was performed separately for each cell line. For quality control plots, the DESeq function was run considering all the RPF or total RNA samples and grouping the data based on shRNA and cell line. The principal component analysis was calculated after variance stabilizing transformation (blind was set to FALSE) using the 500 with the highest row variance and visualized with ggplot2 (*67*). Correlation between samples was calculated with the normalized counts and visualized with corrplot package (*68*).

For transcript-centered analysis of RPFs, the GitHub pipeline from Bushell-Lab (https://github.com/Bushell-lab/Ribo-seq) was followed with minor adaptations. Briefly, the most expressed isoform according to RSEM output in the total RNA data was considered. For the selected isoform, the length of the UTRs and the CDS was extracted from the GTF file of the hg38 reference (Ensembl release 95). RPF reads that were not discarded with ribodetector were re-aligned with bbmap (*69*) with a fasta file containing the sequences only of the most abundant isoform. Then, the number of RPFs per transcript and the position of the RPF in the mRNA were calculated for each read length to calculate the frame, periodicity and P-site offset. For metaprofile analysis, only reads with lengths between 28 and 31 with an offset of 12 or lengths between 32 and 35 with an offset of 13 were considered. Additionally, included transcripts had a minimum length of 20 bp in the 5'UTRs and of 100 in the CDS. Counts of RPFs were normalized according

to the factor size calculated in DESeq2, binned into 25 windows for the UTRs and 50 windows in the CDS, and then averaged across conditions.
The presence of uORFs annotated in the uORF database (*70*) was evaluated by mapping the Ensembl gene IDs to NCBI gene IDs with the R package biomaRT (*71*). If a gene had at least one uORF annotated in the 5'UTR (uORFCDSdistance less than 0) of any category (overlapping or N-terminal extension), it was classified as containing an uORF.

RIP-Seq analysis
The raw RNA-seq data was processed using the nf-core/rnaseq v3.10.1 Nextflow pipeline (*72, 73*). Adaptor trimming was performed with TrimGalore (*65*), reads were aligned to the hg38 genome reference with STAR (*63*) and transcript quantification was done with "RNA-Seq by expectation-Maximization" (RSEM) (*74*).
The DESeq2 (*66*) Bioconductor package in R was used to identify the RNAs enriched in the IP conditions compared to both, the input and the IgG. RNAs with a Log2FoldChange > 1 and p-value < 0.05 in both comparisons were considered eIF2A RNA targets. The contrast between Mel-ST and Mel-STR was also applied. For all contrasts, DESeq2 independent filtering was applied. The principal component analysis was calculated after variance stabilizing transformation (blind was set to FALSE) using the 500 genes with the highest row variance and visualized with ggplot2 (*67*). Correlation between samples was calculated with the normalized counts and visualized with corrplot package (*68*).
For Gene Ontology analysis, the function 'CompareCluster' from the ClusterProfiler Bioconductor R package (*75*) was used considering cell compartment terms.

irCLIP analysis
Data was processed with the nextflow2-based clipseq pipeline from Goodwright (https://github.com/goodwright/clipseq) that trims adaptors with trimGalore (*65*), pre-aligns the reads against tRNA and other small RNA sequences with bowtie (*76*) to then map the unaligned reads against the human hg38 reference with STAR (*63*) and remove duplicated reads with UMI tools (*61*). Finally, crosslinked sites are identified by retrieving the base upstream at the beginning of the read and then peaks obtained with the peak caller clippy. To characterize gene biotypes, the number of deduplicated reads mapped to hg38 reference were counted and classified as protein-coding genes or other gene types (excluding the rRNA 5S subunit). Moreover, trimmed reads were re-aligned to tRNA sequences from gtRNAdb (hg38-tRNAs.fa) (*77*) and rRNA sequences from ncbi (5S, 5.8S, 18S and 28S including predicted sequences, table S9) with bowtie, allowing a maximum of 2 mismatches and reporting only the best alignment. Note that each read was counted only once, even if it mapped to several genes from the same biotype.
To define eIF2A direct RNA targets in SK-Mel147 cells, first, peaks obtained with clippy were overlapped using the intersect function of bedtools (*78*) with genetic coordinates of hg38 reference that belong to CDS or UTRs (stop codon coordinates were included as CDS). Only peaks mapping to these regions were considered. Finally, genes with peaks in at least two replicates were regarded

as direct eIF2A targets. To calculate the density of crosslinked sites in peaks along the CDS and UTRs of targets identified in RIP-seq and irCLIP, only the most expressed isoform calculated for the RIP-seq was considered. For each peak, the R Package GenomicRanges (*79*) was used to calculate the relative position of the peak center in the CDS and in the UTRs separately.

Interactome analysis

Samples were analyzed by LC-MSMS in the Orbitrap Lumos with a 60 min gradient. As a quality control BSA controls were digested in parallel and ran between each of your samples to avoid carryover and assess the instrument performance. Samples were searched against the SwissProt human database using the search algorithm Mascot v2.6. (http://www.matrixscience.com/). Peptides have been filtered based on FDR and only peptides showing an FDR lower than 1% have been retained.

Significance Analysis of INTeractome (SAINT) software package for scoring protein-protein interactions (*80*) was used to remove nonspecific interactions in an unbiased manner and define true interactors by comparing the immunoprecipitated samples to the IgG samples. A BFDR < 0.05 was established to consider a prey as a true interactor. Direct interactors were defined as preys that were immunoprecipitated in both the presence and the absence of RNase. The number of spectral counts in each condition were considered for sample correlation with the R corrplot package (*68*). Principal component analysis of spectral data was calculated with the base R function ‘prcomp’ scaling the samples (R core Team).

The mass spectrometry proteomics data have been deposited to the ProteomeXchange Consortium (http://proteomecentral.proteomexchange.org) via the PRIDE partner repository (*81*) with the dataset identifier “PXD055625”.

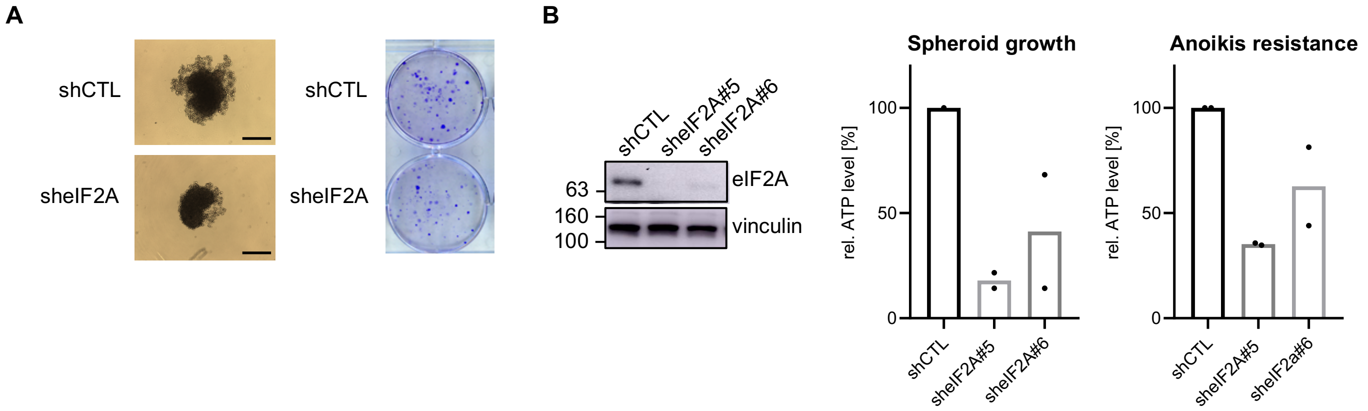

Fig. S1. eIF2A promotes tumoral traits; related to Figure 1
(**A**) Representative images of UACC-62 spheroids (left) and colony plates (right) after eIF2A depletion. Scale bar 500 µm. (**B**) Depletion of eIF2A with additional shRNA hairpins decreases spheroid formation and anoikis resistance. A Western blot showing the efficiency of depletion is shown on the left. Graphs depict the mean of two independent biological replicates, each consisting of at least 5 technical replicates.

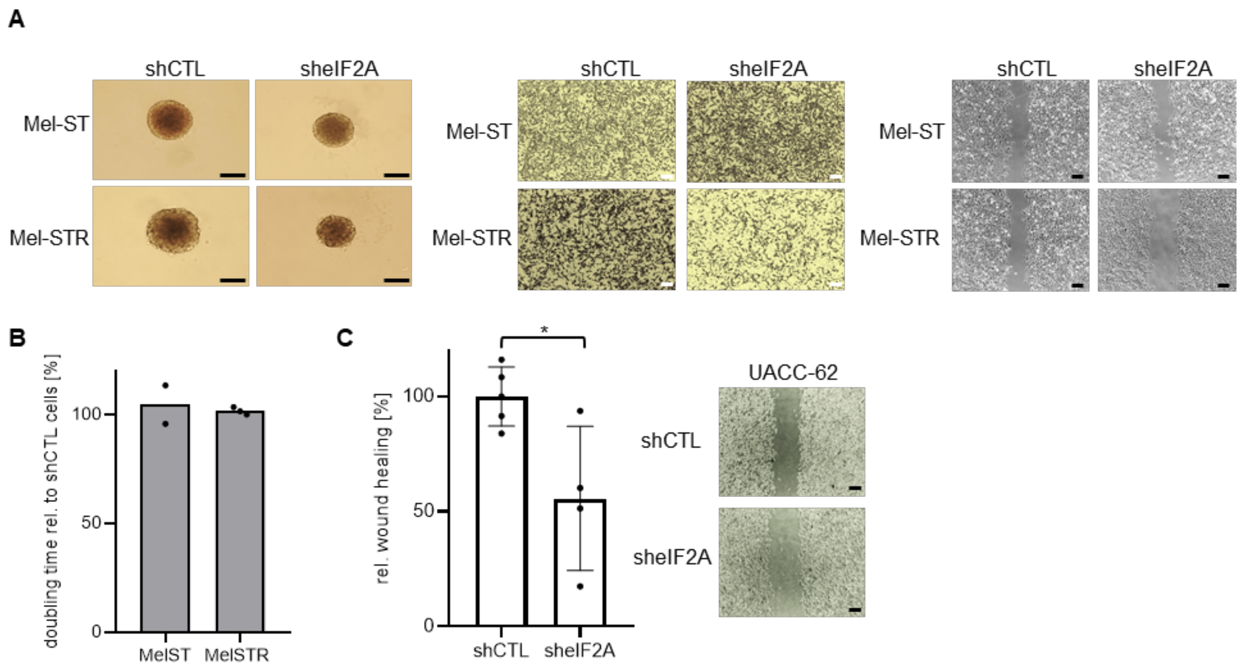

Fig. S2. Depletion of eIF2A affects Mel-STR but not Mel-ST cells; related to Figure 2D

(**A**) Representative images showing the effect of eIF2A depletion on spheroid formation (left), and migration in a transwell assay (middle) or a wound healing assay (right). Scale bar 200 µm. (**B**) eIF2A depletion does not affect proliferation. Bar graphs depict the mean of 2-3 independent experiments. (**C**) Depletion of eIF2A decreases UACC-62 cell migration. Representative images are shown on the right. Bar graphs depict mean + SD of 4 independent replicates. Statistics were calculated using the two-tailed Student´s t-test (* $p < 0.05$).

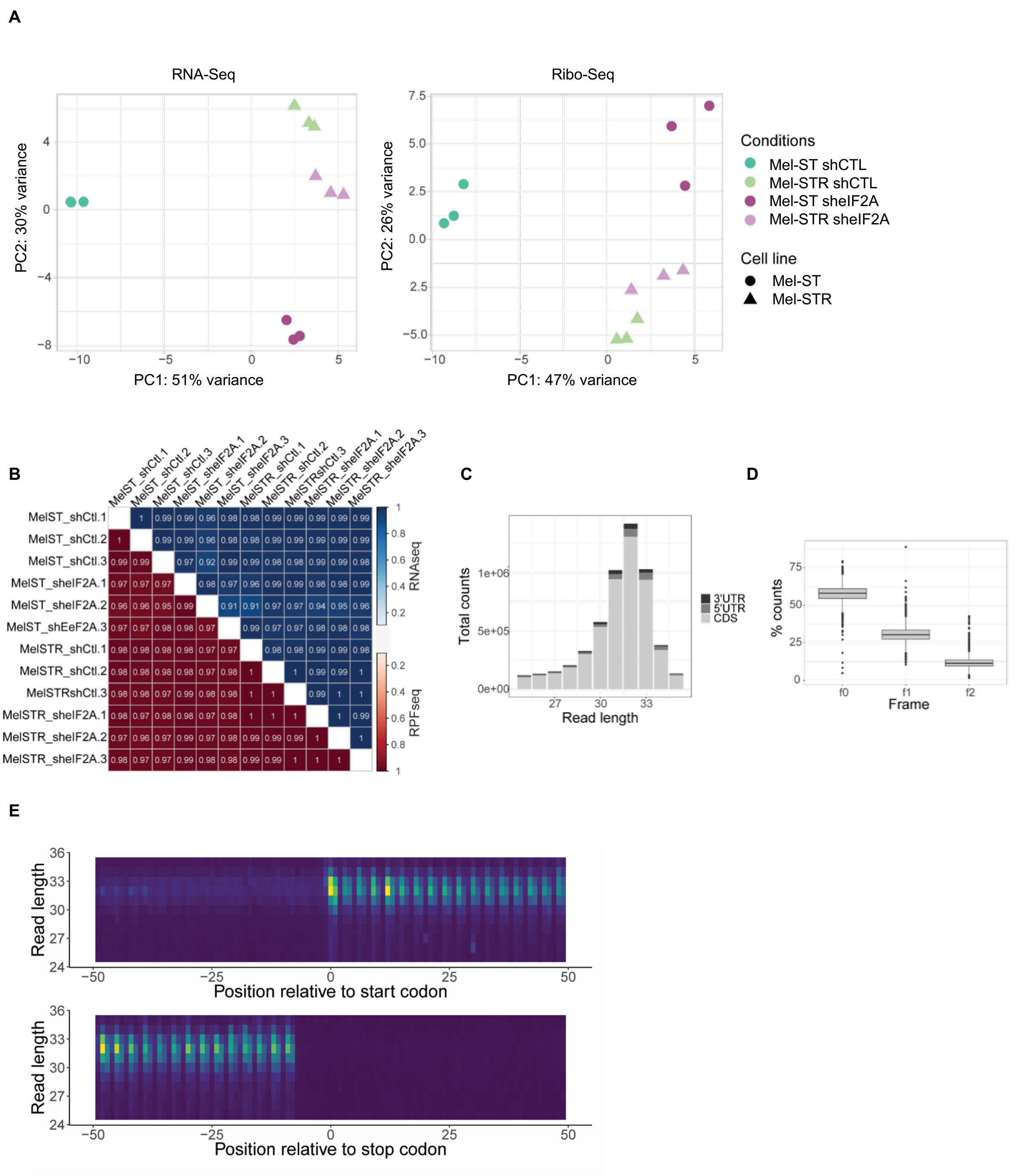

Fig. S3. Ribosome profiling quality control measurements; related to Figure 3

(**A**) Principal Component (PC) analysis of RNA-Seq and Ribo-Seq samples. (**B**) Pairwise Pearson correlation matrix of normalized RNA-Seq (blue) and Ribo-Seq (red) samples. (**C**) Histogram of read lengths obtained for one representative Ribo-Seq sample. (**D**) Percentage of P-site counts in each frame for the 32 nt read length. (**E**) Heatmap depicting the 3-nucleotide periodicity of RPFs. Yellow indicates higher P-site count density.

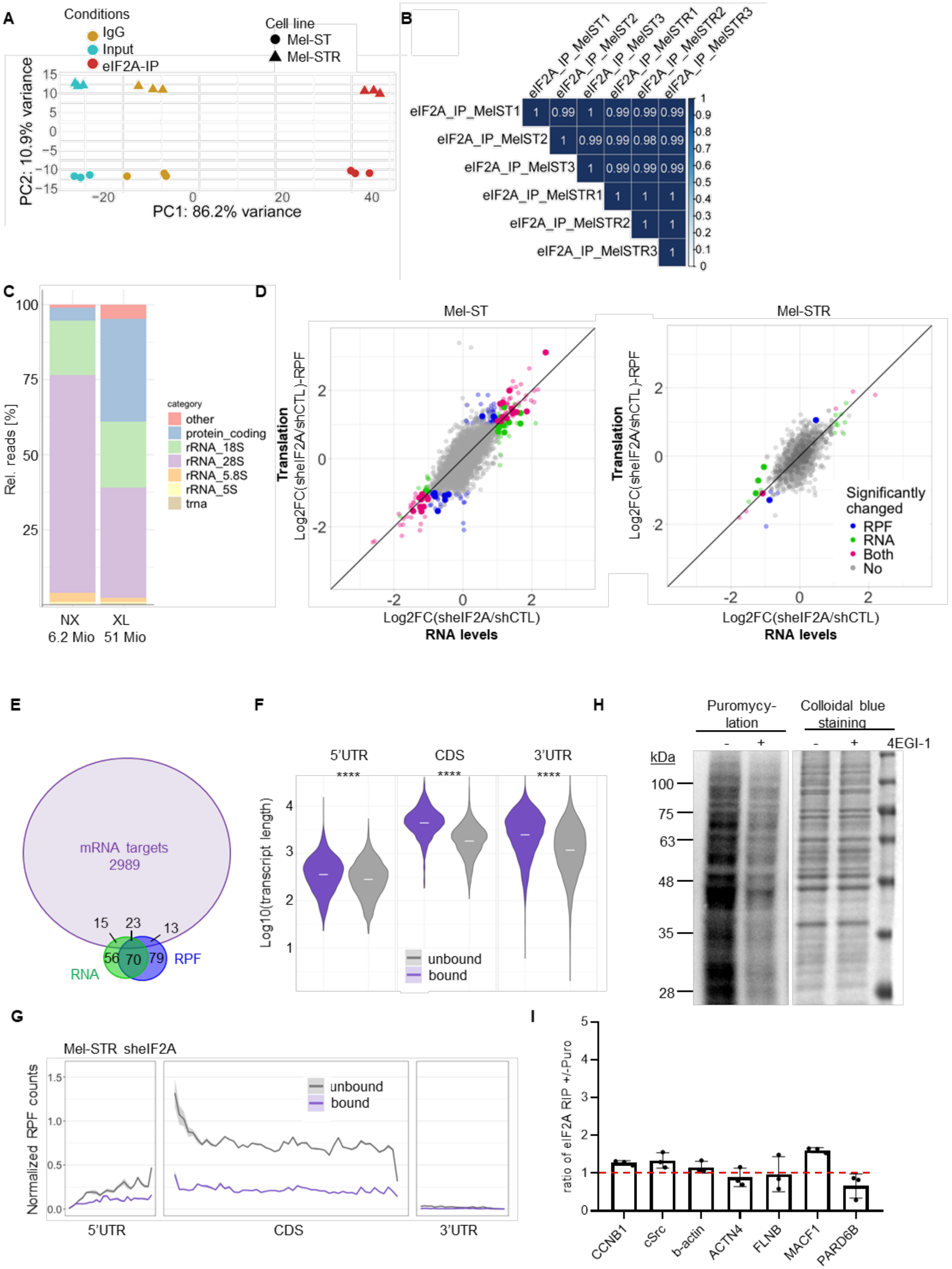

A
Conditions
IgG
Input
eIF2A-IP
Cell line
Mel-ST
Mel-STR
PC2: 10.9% variance
PC1: 86.2% variance
B
eIF2A_IP_MelST1
eIF2A_IP_MelST2
eIF2A_IP_MelST3
eIF2A_IP_MelSTR1
eIF2A_IP_MelSTR2
eIF2A_IP_MelSTR3
C
Rel. reads [%]
category
other
protein_coding
rRNA_18S
rRNA_28S
rRNA_5.8S
rRNA_5S
trna
NX 6.2 Mio
XL 51 Mio
D
Mel-ST
Mel-STR
Translation Log2FC(sheIF2A/shCTL)-RPF
Log2FC(sheIF2A/shCTL) RNA levels
Significantly changed
RPF
RNA
Both
No
E
mRNA targets 2989
15
23
13
56
70
79
RNA
RPF
F
5'UTR
CDS
3'UTR
Log10(transcript length)
unbound
bound
G
Mel-STR sheIF2A
Normalized RPF counts
unbound
bound
5'UTR
CDS
3'UTR
H
Puromycy-lation
Colloidal blue staining
4EGI-1
kDa
100
75
63
48
35
28
I
ratio of eIF2A RIP +/-Puro
CCNB1
cSrc
b-actin
ACTN4
FLNB
MACF1
PARD6B

Fig. S4. Identification and analysis of eIF2A targets; related to Figure 4

(**A**) Principal component analysis of RIP-seq samples. (**B**) Pearson correlation of normalized counts from eIF2A RIP-Seq samples. (**C**) Box chart showing the proportion of RNA biotypes detected by irCLIP in non-crosslinked (NX) and crosslinked samples (XL). Total number of irCLIP reads are indicated below. (**D**) Scatter plot as in Fig. 3B and D with regulated eIF2A targets highlighted by larger and more intense dots. (**E**) Venn diagram showing the overlap between eIF2A RIP-Seq targets (purple) and mRNAs changing at the RPF (blue) or RNA (green) levels upon eIF2A depletion in either Mel-ST or MelSTR. (**F**) Violin plots comparing the length of each transcript region in eIF2A targets and non-targets. Lengths were calculated based on the most expressed isoform. White line represents the mean. Statistics were calculated using the Wilcoxon test (**** $p < 10^{-16}$). (**G**) Metagene analysis of normalized RPF counts in eIF2A targets (purple) and non-targets (grey). Footprint reads were normalized with DESeq2 size factor. (**H**) Puromycylation assay to confirm translational repression by 4EGI-1. Colloidal blue staining is shown to address equal loading. (**I**) RIP-RT/qPCR of eIF2A mRNA targets in Mel-STR cells in the presence or absence of puromycin. Description is as in the legend of Fig. 4I. Statistics were calculated using the two-tailed Student´s t-test (n= 3).

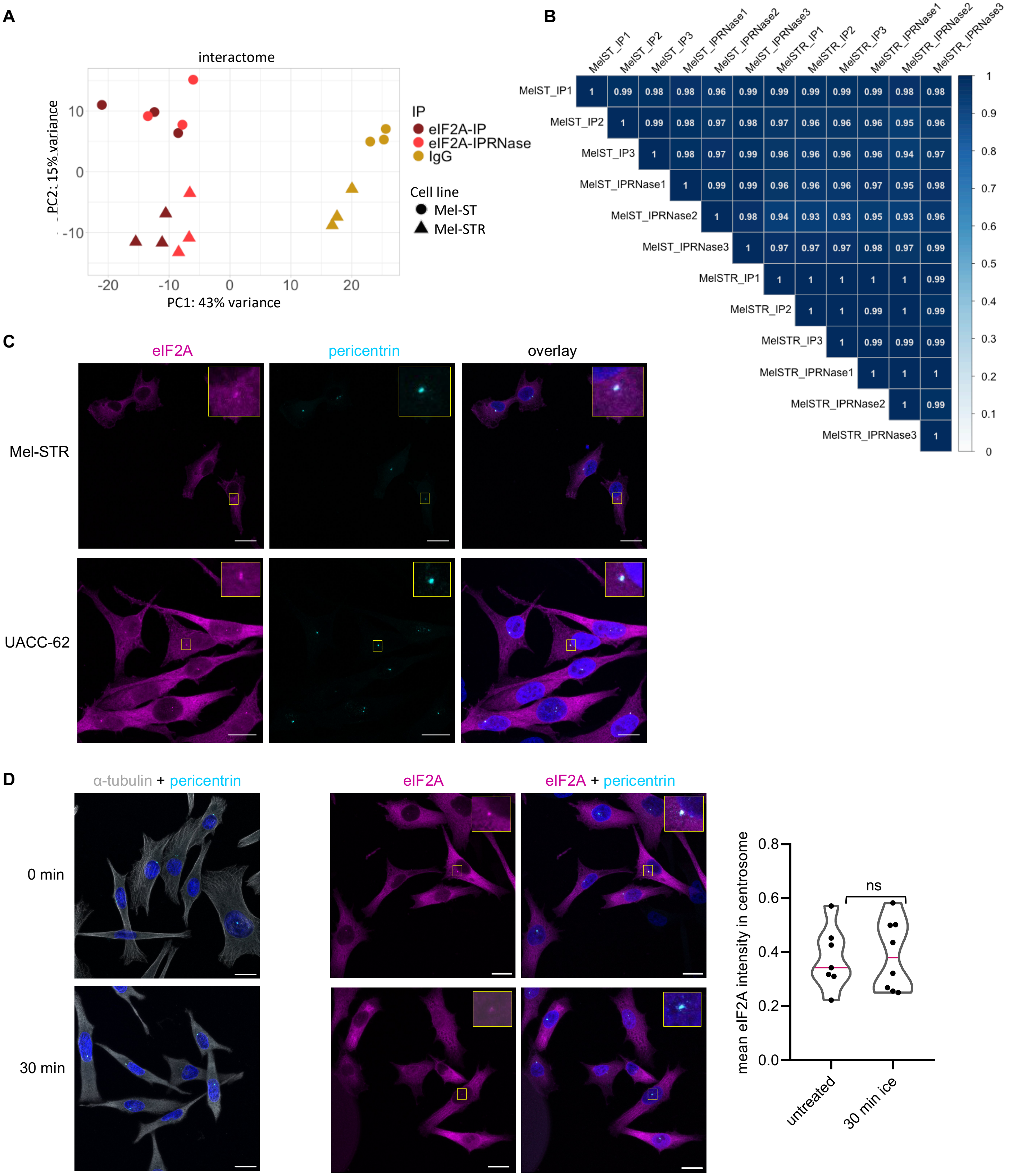
A
interactome
PC2: 15% variance
PC1: 43% variance
10
0
-10
-20
-10
0
10
20
IP
eIF2A-IP
eIF2A-IPRNase
IgG
Cell line
Mel-ST
Mel-STR
B
MelST_IP1
MelST_IP2
MelST_IP3
MelST_IPRNase1
MelST_IPRNase2
MelST_IPRNase3
MelSTR_IP1
MelSTR_IP2
MelSTR_IP3
MelSTR_IPRNase1
MelSTR_IPRNase2
MelSTR_IPRNase3
MelST_IP1 1 0.99 0.98 0.98 0.96 0.99 0.99 0.99 0.99 0.99 0.98 0.98
MelST_IP2 1 0.99 0.98 0.97 0.98 0.97 0.96 0.96 0.96 0.95 0.96
MelST_IP3 1 0.98 0.97 0.99 0.96 0.96 0.96 0.96 0.94 0.97
MelST_IPRNase1 1 0.99 0.99 0.96 0.96 0.96 0.97 0.95 0.98
MelST_IPRNase2 1 0.98 0.94 0.93 0.93 0.95 0.93 0.96
MelST_IPRNase3 1 0.97 0.97 0.97 0.98 0.97 0.99
MelSTR_IP1 1 1 1 1 1 0.99
MelSTR_IP2 1 1 0.99 1 0.99
MelSTR_IP3 1 0.99 0.99 0.99
MelSTR_IPRNase1 1 1 1
MelSTR_IPRNase2 1 0.99
MelSTR_IPRNase3 1
1
0.9
0.8
0.7
0.6
0.5
0.4
0.3
0.2
0.1
0
C
eIF2A
pericentrin
overlay
Mel-STR
UACC-62
D
α-tubulin + pericentrin
eIF2A
eIF2A + pericentrin
0 min
30 min
mean eIF2A intensity in centrosome
0.8
0.6
0.4
0.2
0.0
ns
untreated
30 min ice

Fig. S5. eIF2A localizes to the centrosome; related to Figure 5

(**A**) Principal component analysis of samples used to identify the eIF2A protein interactome. (**B**) Pearson correlation of normalized counts from samples in (A). (**C**) Co-immunofluorescence of eIF2A (magenta) and pericentrin (cyan) in Mel-STR and UACC-62 cells. Insets show magnified views of the centrosome. Scale bar = 20 µm. (**D**) Localization of eIF2A to the centrosome does not depend on microtubule dynamics. Left: 30 min ice treatment depolymerizes microtubules as shown by co-immunofluorescence of α-tubulin (grey) and pericentrin (cyan). Middle: Co-immunofluorescence of eIF2A (magenta) and pericentrin (cyan). Insets show magnified views of the centrosome. Scale bar = 20 µm. Right: Quantification of eIF2A intensity at the centrosome. Each dot in the violin plot reflects the mean of the upper quartile intensity of eIF2A at the centrosome, marked by pericentrin, in 5-10 cells and the red line represents the median. Statistics were calculated using unpaired two-tailed Student´s t-test (n=2); ns, not significant.

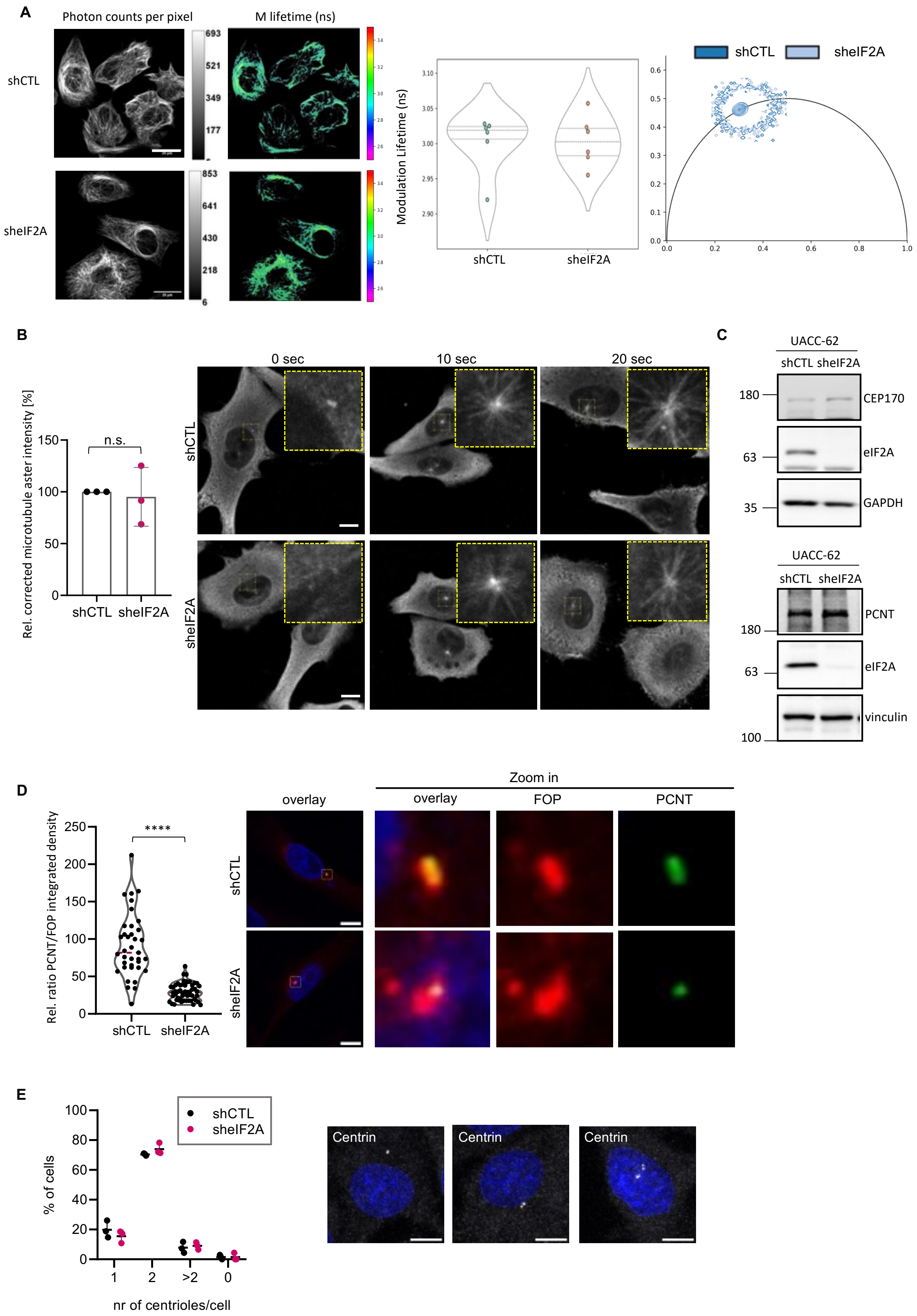

A
Photon counts per pixel
M lifetime (ns)
shCTL
sheIF2A
693
521
349
177
853
641
430
218
6
Modulation Lifetime (ns)
3.10
3.05
3.00
2.95
2.90
shCTL
sheIF2A
shCTL
sheIF2A
0.6
0.5
0.4
0.3
0.2
0.1
0.0
0.0
0.2
0.4
0.6
0.8
1.0
B
Rel. corrected microtubule aster intensity [%]
150
100
50
0
n.s.
shCTL
sheIF2A
0 sec
10 sec
20 sec
shCTL
sheIF2A
C
UACC-62
shCTL sheIF2A
180
CEP170
63
eIF2A
35
GAPDH
UACC-62
shCTL sheIF2A
PCNT
180
63
eIF2A
vinculin
100
D
Rel. ratio PCNT/FOP integrated density
250
200
150
100
50
0
****
shCTL
sheIF2A
overlay
Zoom in
overlay
FOP
PCNT
shCTL
sheIF2A
E
% of cells
100
80
60
40
20
0
shCTL
sheIF2A
1
2
>2
0
nr of centrioles/cell
Centrin
Centrin
Centrin

Fig. S6. eIF2A enforces centrosome composition; related to Figure 6

(A) eIF2A depletion does not affect microtubule stability as measured by FLIM. Left, representative intensity maps showing photon counts per pixel and representative lifetime maps depicting the modulation lifetime (in nanoseconds). Middle, violin plot depicting the distribution of modulation lifetimes. Each dot represents the mean of lifetimes of 2-4 cells. Scale bar = 20 µm. Right, phasor Plot prepared with FLIMPA software of UACC-62 shCTL and sheIF2A cells, with the x- and y-axis representing the G- and S-phasor coordinates. **(B)** Centrosomes efficiently nucleate microtubules after depletion of eIF2A. Microtubules were depolymerized by treatment for 30 min on ice, and microtubule regrowth was allowed to occur at 37º C for the indicated time periods. Insets show enlargements of α-tubulin signal at the centrosome region. Scale bar = 10 µm. **(C)** Global CEP170 and PCNT levels in UACC-62 cells after depletion of eIF2A. **(D)** Violin plot with median depicting the ratio of PCNT to FOP integrated densities measured inside the same centrosome. Each dot represents the mean ratio of 5-15 cells (n= 3, Student´s t test: **** $p < 0.0001$). Representative images with magnification of the centrosome are shown on the right. Scale bar = 10 µm. **(E)** Number of centrioles per cell based on Centrin staining (n= 3). Each dot represents the average of at least 30 cells. Representative images of cells containing one, two or more than two centrioles are shown on the right. Scale bar = 5 µm.

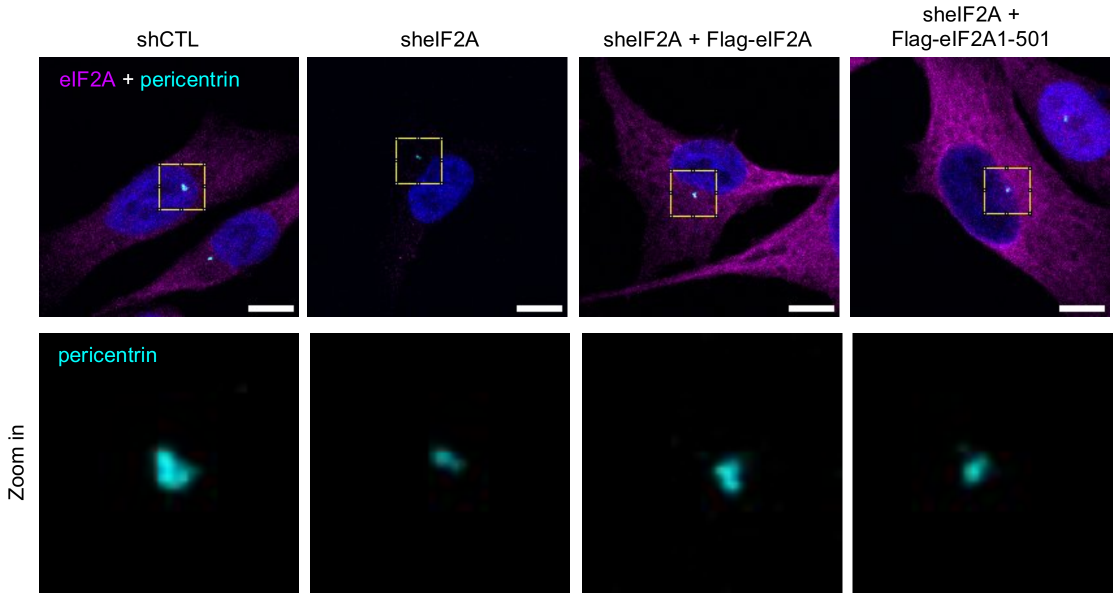

Fig. S7. eIF2A enforces centrosome composition via its RNA-binding activity; related to Figure 7.
Co-immunofluorescence of eIF2A (magenta) and pericentrin (cyan) in UACC-62 cells. Scale bar = 10 µm. Magnifications in the lower row show centrosome region.

Supplementary Tables:

Table S1. (separate file)

Genes regulated by eIF2A. Tab1: Ribo-seq and RNA-seq log2 Fold Changes (LFC) between sheIF2A and shCTL in Mel-STR and Mel-ST cells. Genes with a LFC > |1| and an adjusted p-value < 0.05 were considered regulated (padj_log2FoldChange column).

Table S2. (separate file)

eIF2A mRNA targets. Log2 Fold Changes (LFC) between anti-eIF2A RIP and either anti-IgG RIP or input in Mel-ST and Mel-STR cells. mRNAs with a LFC > 1 and an adjusted p-value < 0.05 in both comparisons in at least one cell line are considered eIF2A targets. LFC and significance of differences between Mel-STR and Mel-ST cells are also included.

Table S3. (separate file)

Cell compartment gene ontology terms of eIF2A targets. Output table from compareCluster function of clusterProfiler Bioconductor R package.

Table S4. (separate file)

irCLIP peaks of eIF2A targets in SK-MEL-147 cells. Transcripts are considered to be targets of eIF2A if they have peaks in the CDS and/or UTR regions in at least two replicates. The crosslinked counts of each peak are shown for each replicate. Peaks in genes that are also RIP-Seq targets are indicated.

Table S5. (separate file)

eIF2A protein interactors. Output table from SAINT in Mel-STR and Mel-ST cells. Under the bait column, conditions of immunoprecipitation are indicated. Spec shows spectral counts from the anti-eIF2A IP, and Ctl-Counts from the IgG control.

Table S6. (separate file)

Cell compartment gene ontology terms of eIF2A interactors. Output table from compareCluster function of clusterProfiler Bioconductor R package.

Table S7.

qPCR primers

gene	forward	reverse
CCNB1	AGGCGAAGATCAACATGGCA	AGCTGTTCTTGGCCTCAGTC
GAPDH	TGCACCACCAACTGCTTAG	GATGCAGGGATGATGTTC
cSrc	CAGTGTCTGACTTCGACAACGC	CCATCGGCGTGTTTGGAGTA
b-actin	CCTTTGCCGATCCGCCGC	CCATCACGCCCTGGTGCC
ACTN4	CAACCACTTCGACAAGAAGCAG	AGATAAGCAGAGCCCTGAAGTC
FLNB	TGTCATGGTGACCGAAGAGG	AGGCCGTTCATGTCACTCAC
MACF1	AAGCTGGCCCCGAATGAAG	CTCCCTAGCTGGGGTGTTTG
ARHGAP6	AGAGTGCCAGGGCAGTTTATG	GCTCCAGTTACCTTTCCCAAG
PARD6B	ACGGCCAATCCACTGCTTAG	CCAAAGGCACTGTAGTCTGC
PTPRU	GGGCTTATCCTGGGCATCTG	TAGTGGTCTCTCCCTTTGCG
Nin	GGAGACACGCTACGATGAGG	CAGCTCATCCTGCAACCTGA
Numa1	GACCTCTGAGCTCACCACAC	GCCAGCCAGTTCTTGATCCT
Bicd2	TGGGGACTACTACGAGGTGG	TTGCAGGCCAAGATCTCAGG
PCNT	GCAAACCTCCTCTCCATGCT	CTCCAAACAAACCCAGCAGC
CEP350	GGTTGCAGCAAGAAAAGGCA	GCTGTCTTTCCTTCCGAGCT
CCP110	AGCTGAGCAGGAAAGGGAAC	TCCGCTGTCATTGCCTTCTT
GPSM1	CCAAGAGCAGGGAGACAAGG	GGACAGTTGCTTGCCTTTGG
CDR2L	TATTGGGATGGGGCTGAGGA	GGAGGTGAAGAGCAGGCATT
CDK5RAP2	CAGAGAACCACAGACTGCGT	AGTCTCTCCCTCTCCTGCTG

Table S8.

irCLIP_ddRT primers

Sample	primer	Primer sequence*
Non-xl 1	irCLIP_ddRT_13	/5Phos/ WWW TCCGG NNNN AGATCGGAAGAGCGTCGTGAT

		/iSp18/ GGATCC /iSp18/ TACTGAACCGC
Non-xl 2	irCLIP_ddRT_14	/5Phos/ WWW TGCCT NNNN AGATCGGAAGAGCGTCGTGAT /iSp18/ GGATCC /iSp18/ TACTGAACCGC
Non-xl 3	irCLIP_ddRT_15	/5Phos/ WWW TATTC NNNN AGATCGGAAGAGCGTCGTGAT /iSp18/ GGATCC /iSp18/ TACTGAACCGC
Xli 1	irCLIP_ddRT_17	/5Phos/ WWW AAATG NNNN AGATCGGAAGAGCGTCGTGAT /iSp18/ GGATCC /iSp18/ TACTGAACCGC
Xli 2	irCLIP_ddRT_18	/5Phos/ WWW AAGGT NNNN AGATCGGAAGAGCGTCGTGAT /iSp18/ GGATCC /iSp18/ TACTGAACCGC
Xli 4	irCLIP_ddRT_20	/5Phos/ WWW ACGCA NNNN AGATCGGAAGAGCGTCGTGAT /iSp18/ GGATCC /iSp18/ TACTGAACCGC

* WWW, 3 wobble mix of A dn T; N, any nucleotide; iSP18, 18-atom hexaethyleneglycol spacer

Table S9. (separate file)
NCBI transcript IDs of human rRNA used in the iCLIP analysis.

Table S10. (separate file)
List of used smFISH probes and their sequences.

Movie S1. (separate file)
Related to Figure 5, this movie shows the colocalization of eIF2A (red) with the centrosomal protein CEP170 (green) in Mel-STR cells.

REFERENCES AND NOTES

1. M. G. Kearse, J. E. Wilusz, Non-AUG translation: A new start for protein synthesis in eukaryotes. *Genes Dev.* **31**, 1717–1731 (2017).

2. R. Lacerda, J. Menezes, L. Romao, More than just scanning: The importance of cap-independent mRNA translation initiation for cellular stress response and cancer. *Cell. Mol. Life Sci.* **74**, 1659–1680 (2017).

3. A. Sendoel, J. G. Dunn, E. H. Rodriguez, S. Naik, N. C. Gomez, B. Hurwitz, J. Levorse, B. D. Dill, D. Schramek, H. Molina, J. S. Weissman, E. Fuchs, Translation from unconventional 5′ start sites drives tumour initiation. *Nature* **541**, 494–499 (2017).

4. S. R. Starck, V. Jiang, M. Pavon-Eternod, S. Prasad, B. McCarthy, T. Pan, N. Shastri, Leucine-tRNA initiates at CUG start codons for protein synthesis and presentation by MHC class I. *Science* **336**, 1719–1723 (2012).

5. S. R. Starck, J. C. Tsai, K. Chen, M. Shodiya, L. Wang, K. Yahiro, M. Martins-Green, N. Shastri, P. Walter, Translation from the 5′ untranslated region shapes the integrated stress response. *Science* **351**, aad3867 (2016).

6. E. Kim, J. H. Kim, K. Seo, K. Y. Hong, S. W. A. An, J. Kwon, S. V. Lee, S. K. Jang, eIF2A, an initiator tRNA carrier refractory to eIF2alpha kinases, functions synergistically with eIF5B. *Cell. Mol. Life Sci.* **75**, 4287–4300 (2018).

7. J. H. Kim, S. M. Park, J. H. Park, S. J. Keum, S. K. Jang, eIF2A mediates translation of hepatitis C viral mRNA under stress conditions. *EMBO J.* **30**, 2454–2464 (2011).

8. O. S. Kwon, S. An, E. Kim, J. Yu, K. Y. Hong, J. S. Lee, S. K. Jang, An mRNA-specific tRNAi carrier eIF2A plays a pivotal role in cell proliferation under stress conditions: Stress-resistant translation of c-Src mRNA is mediated by eIF2A. *Nucleic Acids Res.* **45**, 296–310 (2017).

9. I. Ventoso, M. A. Sanz, S. Molina, J. J. Berlanga, L. Carrasco, M. Esteban, Translational resistance of late alphavirus mRNA to eIF2alpha phosphorylation: A strategy to overcome the antiviral effect of protein kinase PKR. *Genes Dev.* **20**, 87–100 (2006).

10. H. Kim, D. Aponte-Diaz, M. S. Sotoudegan, D. Shengjuler, J. J. Arnold, C. E. Cameron, The enterovirus genome can be translated in an IRES-independent manner that requires the initiation factors eIF2A/eIF2D. *PLOS Biol.* **21**, e3001693 (2023).

11. A. A. Komar, W. C. Merrick, A retrospective on eIF2A—and not the alpha subunit of eIF2. *Int. J. Mol. Sci.* **21**, 2054 (2020).

12. E. Gonzalez-Almela, H. Williams, M. A. Sanz, L. Carrasco, The initiation factors eIF2, eIF2A, eIF2D, eIF4A, and eIF4G are not involved in translation driven by hepatitis C virus IRES in human cells. *Front. Microbiol.* **9**, 207 (2018).

13. Z. A. Jaafar, A. Oguro, Y. Nakamura, J. S. Kieft, Translation initiation by the hepatitis C virus IRES requires eIF1A and ribosomal complex remodeling. *eLife* **5**, e21198 (2016).

14. M. A. Sanz, E. Gonzalez Almela, L. Carrasco, Translation of Sindbis subgenomic mRNA is independent of eIF2, eIF2A and eIF2D. *Sci. Rep.* **7**, 43876 (2017).

15. K. Ichihara, A. Matsumoto, H. Nishida, Y. Kito, H. Shimizu, Y. Shichino, S. Iwasaki, K. Imami, Y. Ishihama, K. I. Nakayama, Combinatorial analysis of translation dynamics reveals eIF2 dependence of translation initiation at near-cognate codons. *Nucleic Acids Res.* **49**, 7298–7317 (2021).

16. S. Gaikwad, F. Ghobakhlou, H. Zhang, A. G. Hinnebusch, Yeast eIF2A has a minimal role in translation initiation and uORF-mediated translational control in vivo. *eLife* **12**, RP92916 (2024).

17. D. J. Grove, D. J. Levine, M. G. Kearse, Increased levels of eIF2A inhibit translation by sequestering 40S ribosomal subunits. *Nucleic Acids Res.* **51**, 9983–10000 (2023).

18. L. Chen, J. He, J. Zhou, Z. Xiao, N. Ding, Y. Duan, W. Li, L. Q. Sun, EIF2A promotes cell survival during paclitaxel treatment in vitro and in vivo. *J. Cell. Mol. Med.* **23**, 6060–6071 (2019).

19. P. Xia, H. Zhang, K. Xu, X. Jiang, M. Gao, G. Wang, Y. Liu, Y. Yao, X. Chen, W. Ma, Z. Zhang, Y. Yuan, MYC-targeted WDR4 promotes proliferation, metastasis, and sorafenib

resistance by inducing CCNB1 translation in hepatocellular carcinoma. *Cell Death Dis.* **12**, 691 (2021).

20. H. Liang, S. He, J. Yang, X. Jia, P. Wang, X. Chen, Z. Zhang, X. Zou, M. A. McNutt, W. H. Shen, Y. Yin, PTENalpha, a PTEN isoform translated through alternative initiation, regulates mitochondrial function and energy metabolism. *Cell Metab.* **19**, 836–848 (2014).

21. D. Schadendorf, A. C. J. van Akkooi, C. Berking, K. G. Griewank, R. Gutzmer, A. Hauschild, A. Stang, A. Roesch, S. Ugurel, Melanoma. *Lancet* **392**, 971–984 (2018).

22. B. I. Ratnikov, D. A. Scott, A. L. Osterman, J. W. Smith, Z. A. Ronai, Metabolic rewiring in melanoma. *Oncogene* **36**, 147–157 (2017).

23. N. Mestre-Farras, S. Guerrero, N. Bley, E. Rivero, O. Coll, E. Borras, E. Sabido, A. Indacochea, C. Casillas-Serra, A. I. Jarvelin, B. Oliva, A. Castello, S. Huttelmaier, F. Gebauer, Melanoma RBPome identification reveals PDIA6 as an unconventional RNA-binding protein involved in metastasis. *Nucleic Acids Res.* **50**, 8207–8225 (2022).

24. P. B. Gupta, C. Kuperwasser, J. P. Brunet, S. Ramaswamy, W. L. Kuo, J. W. Gray, S. P. Naber, R. A. Weinberg, The melanocyte differentiation program predisposes to metastasis after neoplastic transformation. *Nat. Genet.* **37**, 1047–1054 (2005).

25. N. T. Ingolia, G. A. Brar, S. Rouskin, A. M. McGeachy, J. S. Weissman, The ribosome profiling strategy for monitoring translation in vivo by deep sequencing of ribosome-protected mRNA fragments. *Nat. Protoc.* **7**, 1534–1550 (2012).

26. L. H. Wei, Y. Sun, J. U. Guo, Genome-wide CRISPR screens identify noncanonical translation factor eIF2A as an enhancer of SARS-CoV-2 programmed-1 ribosomal frameshifting. *Cell Rep.* **42**, 112987 (2023).

27. N. J. Moerke, H. Aktas, H. Chen, S. Cantel, M. Y. Reibarkh, A. Fahmy, J. D. Gross, A. Degterev, J. Yuan, M. Chorev, J. A. Halperin, G. Wagner, Small-molecule inhibition of the interaction between the translation initiation factors eIF4E and eIF4G. *Cell* **128**, 257–267 (2007).

28. M. Bornens, Centrosome organization and functions. *Curr. Opin. Struct. Biol.* **66**, 199–206 (2021).

29. J. Paz, J. Luders, Microtubule-organizing centers: Towards a minimal parts list. *Trends Cell Biol.* **28**, 176–187 (2018).

30. S. Kapsiani, N. F. Läubli, E. N. Ward, M. Shehata, C. F. Kaminski, G. S. Kaminski Schierle, FLIMPA: A versatile software for fluorescence lifetime imaging microscopy phasor analysis. *Anal. Chem.* **97**, 11382–11387 (2024).

31. A. V. Burakov, E. S. Nadezhdina, Centering and shifting of centrosomes in cells. *Cells* **9**, 1351 (2020).

32. J. Elric, S. Etienne-Manneville, Centrosome positioning in polarized cells: Common themes and variations. *Exp. Cell Res.* **328**, 240–248 (2014).

33. R. Chouaib, A. Safieddine, X. Pichon, A. Imbert, O. S. Kwon, A. Samacoits, A. M. Traboulsi, M. C. Robert, N. Tsanov, E. Coleno, I. Poser, C. Zimmer, A. Hyman, H. Le Hir, K. Zibara, M. Peter, F. Mueller, T. Walter, E. Bertrand, A dual protein-mRNA localization screen reveals compartmentalized translation and widespread co-translational RNA targeting. *Dev. Cell* **54**, 773–791.e5 (2020).

34. D. A. Lerit, Signed, sealed, and delivered: RNA localization and translation at centrosomes. *Mol. Biol. Cell* **33**, pe3 (2022).

35. A. Safieddine, E. Coleno, S. Salloum, A. Imbert, A. M. Traboulsi, O. S. Kwon, F. Lionneton, V. Georget, M. C. Robert, T. Gostan, C. H. Lecellier, R. Chouaib, X. Pichon, H. Le Hir, K. Zibara, F. Mueller, T. Walter, M. Peter, E. Bertrand, A choreography of centrosomal mRNAs reveals a conserved localization mechanism involving active polysome transport. *Nat. Commun.* **12**, 1352 (2021).

36. G. Sepulveda, M. Antkowiak, I. Brust-Mascher, K. Mahe, T. Ou, N. M. Castro, L. N. Christensen, L. Cheung, X. Jiang, D. Yoon, B. Huang, L. E. Jao, Co-translational protein

targeting facilitates centrosomal recruitment of PCNT during centrosome maturation in vertebrates. *Elife* **7**, e34959 (2018).

37. D. Iaconis, M. Monti, M. Renda, A. van Koppen, R. Tammaro, M. Chiaravalli, F. Cozzolino, P. Pignata, C. Crina, P. Pucci, A. Boletta, V. Belcastro, R. H. Giles, E. M. Surace, S. Gallo, M. Pende, B. Franco, The centrosomal OFD1 protein interacts with the translation machinery and regulates the synthesis of specific targets. *Sci. Rep.* **7**, 1224 (2017).

38. K. Kashiwagi, T. Ito, S. Yokoyama, Crystal structure of the eukaryotic translation initiation factor 2A from *Schizosaccharomyces pombe*. *J. Struct. Funct. Genomics* **15**, 125–130 (2014).

39. J. Fang, W. Tian, M. A. Quintanilla, J. R. Beach, D. A. Lerit, The PCM scaffold enables RNA localization to centrosomes. *bioRxiv* 2024.01.13.575509, (2024).

40. H. Zein-Sabatto, D. A. Lerit, The identification and functional analysis of mRNA localizing to centrosomes. *Front. Cell Dev. Biol.* **9**, 782802 (2021).

41. J. J. Bravo-Cordero, L. Hodgson, J. Condeelis, Directed cell invasion and migration during metastasis. *Curr. Opin. Cell Biol.* **24**, 277–283 (2012).

42. J. Fares, M. Y. Fares, H. H. Khachfe, H. A. Salhab, Y. Fares, Molecular principles of metastasis: A hallmark of cancer revisited. *Signal Transduct. Target. Ther.* **5**, 28 (2020).

43. S. Etienne-Manneville, Microtubules in cell migration. *Annu. Rev. Cell Dev. Biol.* **29**, 471–499 (2013).

44. C. Garcin, A. Straube, Microtubules in cell migration. *Essays Biochem.* **63**, 509–520 (2019).

45. M. R. Hannaford, N. M. Rusan, Positioning centrioles and centrosomes. *J. Cell Biol.* **223**, e202311140 (2024).

46. J. B. Woodruff, B. Ferreira Gomes, P. O. Widlund, J. Mahamid, A. Honigmann, A. A. Hyman, The centrosome is a selective condensate that nucleates microtubules by concentrating tubulin. *Cell* **169**, 1066–1077.e10 (2017).

47. J. B. Woodruff, A. A. Hyman, E. Boke, Organization and function of non-dynamic biomolecular condensates. *Trends Biochem. Sci.* **43**, 81–94 (2018).

48. M. J. Rale, R. S. Kadzik, S. Petry, Phase transitioning the centrosome into a microtubule nucleator. *Biochemistry* **57**, 30–37 (2018).

49. W. Borcherds, A. Bremer, M. B. Borgia, T. Mittag, How do intrinsically disordered protein regions encode a driving force for liquid-liquid phase separation? *Curr. Opin. Struct. Biol.* **67**, 41–50 (2021).

50. J. Zhao, Y. Li, C. Wang, H. Zhang, H. Zhang, B. Jiang, X. Guo, X. Song, IRESbase: A comprehensive database of experimentally validated internal ribosome entry sites. *Genom. Proteom. Bioinform.* **18**, 129–139 (2020).

51. A. Suarez-Arnedo, F. Torres Figueroa, C. Clavijo, P. Arbelaez, J. C. Cruz, C. Munoz-Camargo, An image J plugin for the high throughput image analysis of in vitro scratch wound healing assays. *PLOS ONE* **15**, e0232565 (2020).

52. J. Jungfleisch, R. Bottcher, M. Tallo-Parra, G. Perez-Vilaro, A. Merits, E. M. Novoa, J. Diez, CHIKV infection reprograms codon optimality to favor viral RNA translation by altering the tRNA epitranscriptome. *Nat. Commun.* **13**, 4725 (2022).

53. R. Avolio, M. Ingles-Ferrandiz, A. Ciocia, O. Coll, S. Bonnin, T. Guitart, A. Ribo, F. Gebauer, Coordinated post-transcriptional control of oncogene-induced senescence by UNR/CSDE1. *Cell Rep.* **38**, 110211 (2022).

54. A. Ezquerra, R. Viais, J. Luders, Assaying microtubule nucleation. *Methods Mol. Biol.* **2101**, 163–178 (2020).

55. C. A. Schneider, W. S. Rasband, K. W. Eliceiri, NIH image to ImageJ: 25 years of image analysis. *Nat. Methods* **9**, 671–675 (2012).

56. N. Tsanov, A. Samacoits, R. Chouaib, A. M. Traboulsi, T. Gostan, C. Weber, C. Zimmer, K. Zibara, T. Walter, M. Peter, E. Bertrand, F. Mueller, smiFISH and FISH-quant - A flexible

single RNA detection approach with super-resolution capability. *Nucleic Acids Res.* **44**, e165 (2016).

57. A. Safieddine, E. Coleno, F. Lionneton, A. M. Traboulsi, S. Salloum, C. H. Lecellier, T. Gostan, V. Georget, C. Hassen-Khodja, A. Imbert, F. Mueller, T. Walter, M. Peter, E. Bertrand, HT-smFISH: A cost-effective and flexible workflow for high-throughput single-molecule RNA imaging. *Nat. Protoc.* **18**, 157–187 (2023).

58. C. Stringer, T. Wang, M. Michaelos, M. Pachitariu, Cellpose: A generalist algorithm for cellular segmentation. *Nat. Methods* **18**, 100–106 (2021).

59. A. Imbert, W. Ouyang, A. Safieddine, E. Coleno, C. Zimmer, E. Bertrand, T. Walter, F. Mueller, FISH-quant v2: A scalable and modular tool for smFISH image analysis. *RNA* **28**, 786–795 (2022).

60. M. Martin, Cutadapt removes adapter sequences from high-throughput sequencing reads. *EMBnet.journal* **17**, 10–12 (2011).

61. T. Smith, A. Heger, I. Sudbery, UMI-tools: Modeling sequencing errors in Unique Molecular Identifiers to improve quantification accuracy. *Genome Res.* **27**, 491–499 (2017).

62. Z. L. Deng, P. C. Munch, R. Mreches, A. C. McHardy, Rapid and accurate identification of ribosomal RNA sequences via deep learning. *Nucleic Acids Res.* **50**, e60 (2022).

63. A. Dobin, C. A. Davis, F. Schlesinger, J. Drenkow, C. Zaleski, S. Jha, P. Batut, M. Chaisson, T. R. Gingeras, STAR: Ultrafast universal RNA-seq aligner. *Bioinformatics* **29**, 15–21 (2013).

64. Y. Liao, G. K. Smyth, W. Shi, featureCounts: An efficient general purpose program for assigning sequence reads to genomic features. *Bioinformatics* **30**, 923–930 (2014).

65. F. J. Krueger, F. James, P. Ewels, E. Afyounian, B. Schuster-Boeckler, FelixKrueger/TrimGalore: v0.6.7. Hawaii: Zenodo (2021); https://zenodo.org/records/5127899.

66. M. I. Love, W. Huber, S. Anders, Moderated estimation of fold change and dispersion for RNA-seq data with DESeq2. *Genome Biol.* **15**, 550 (2014).

67. H. Wickham, ggplot2: Elegant Graphics for Data Analysis (Springer-Verlag, 2016), pp. 1–260.

68. M. Hahsler, K. Hornik, C. Buchta, Getting things in order: An introduction to the R package seriation. *J. Stat. Softw.* **25**, 1–34 (2008).

69. B. Bushnell, BBMap: A Fast, Accurate, Splice-Aware Aligner. *Ernest Orlando Lawrence Berkeley National Library*, Berkeley, CA, (2014). https://sourceforge.net/proects/bbmap/.

70. F. Manske, L. Ogoniak, L. Jurgens, N. Grundmann, W. Makalowski, K. Wethmar, The new uORFdb: Integrating literature, sequence, and variation data in a central hub for uORF research. *Nucleic Acids Res.* **51**, D328–D336 (2023).

71. S. Durinck, Y. Moreau, A. Kasprzyk, S. Davis, B. De Moor, A. Brazma, W. Huber, BioMart and bioconductor: A powerful link between biological databases and microarray data analysis. *Bioinformatics* **21**, 3439–3440 (2005).

72. P. A. Ewels, A. Peltzer, S. Fillinger, H. Patel, J. Alneberg, A. Wilm, M. U. Garcia, P. Di Tommaso, S. Nahnsen, The nf-core framework for community-curated bioinformatics pipelines. *Nat. Biotechnol.* **38**, 276–278 (2020).

73. H. E. Patel, A. Peltzer, R. Hammarén, O. Botvinnik, G. Sturm, nf-core/rnaseq v3.0—Silver Shark (2020). https://zenodo.org/records/4323183.

74. B. Li, C. N. Dewey, RSEM: Accurate transcript quantification from RNA-Seq data with or without a reference genome. *BMC Bioinformatics* **12**, 323 (2011).

75. G. Yu, L. G. Wang, Y. Han, Q. Y. He, clusterProfiler: An R package for comparing biological themes among gene clusters. *OMICS* **16**, 284–287 (2012).

76. B. Langmead, M. C. Schatz, J. Lin, M. Pop, S. L. Salzberg, Searching for SNPs with cloud computing. *Genome Biol.* **10**, R134 (2009).

77. P. P. Chan, T. M. Lowe, GtRNAdb 2.0: An expanded database of transfer RNA genes identified in complete and draft genomes. *Nucleic Acids Res.* **44**, D184–D189 (2016).

78. A. R. Quinlan, I. M. Hall, BEDTools: A flexible suite of utilities for comparing genomic features. *Bioinformatics* **26**, 841–842 (2010).

79. M. Lawrence, W. Huber, H. Pages, P. Aboyoun, M. Carlson, R. Gentleman, M. T. Morgan, V. J. Carey, Software for computing and annotating genomic ranges. *PLoS Comput. Biol.* **9**, e1003118 (2013).

80. H. Choi, B. Larsen, Z. Y. Lin, A. Breitkreutz, D. Mellacheruvu, D. Fermin, Z. S. Qin, M. Tyers, A. C. Gingras, A. I. Nesvizhskii, SAINT: Probabilistic scoring of affinity purification-mass spectrometry data. *Nat. Methods* **8**, 70–73 (2011).

81. J. A. Vizcaino, R. G. Cote, A. Csordas, J. A. Dianes, A. Fabregat, J. M. Foster, J. Griss, E. Alpi, M. Birim, J. Contell, G. O'Kelly, A. Schoenegger, D. Ovelleiro, Y. Perez-Riverol, F. Reisinger, D. Rios, R. Wang, H. Hermjakob, The PRoteomics IDEntifications (PRIDE) database and associated tools: Status in 2013. *Nucleic Acids Res.* **41**, D1063–D1069 (2012).
